# Embryonic neurogenesis in *Pseudopallene* sp. (Arthropoda, Pycnogonida) includes two subsequent phases with similarities to different arthropod groups

**DOI:** 10.1186/2041-9139-4-32

**Published:** 2013-11-29

**Authors:** Georg Brenneis, Angelika Stollewerk, Gerhard Scholtz

**Affiliations:** 1 Humboldt-Universität zu Berlin, Institut für Biologie/Vergleichende Zoologie, Philippstraße 13, Berlin 10115, Germany; 2 School of Biological and Chemical Sciences, Queen Mary University of London, Mile End Road, London E1 4NS, UK

**Keywords:** Development, Evolution, Nervous system, Neural precursor, Sea spiders, Stem cell

## Abstract

**Background:**

Studies on early neurogenesis have had considerable impact on the discussion of the phylogenetic relationships of arthropods, having revealed striking similarities and differences between the major lineages. In Hexapoda and crustaceans, neurogenesis involves the neuroblast, a type of neural stem cell. In each hemi-segment, a set of neuroblasts produces neural cells by repeated asymmetrical and interiorly directed divisions. In Euchelicerata and Myriapoda, neurogenesis lacks neural stem cells, featuring instead direct immigration of neural cell groups from fixed sites in the neuroectoderm. Accordingly, neural stem cells were hitherto assumed to be an evolutionary novelty of the Tetraconata (Hexapoda + crustaceans). To further test this hypothesis, we investigated neurogenesis in Pycnogonida, or sea spiders, a group of marine arthropods with close affinities to euchelicerates.

**Results:**

We studied neurogenesis during embryonic development of *Pseudopallene* sp. (Callipallenidae), using fluorescent histochemical staining and immunolabelling. Embryonic neurogenesis has two phases. The first phase shows notable similarities to euchelicerates and myriapods. These include i) the lack of morphologically different cell types in the neuroectoderm; ii) the formation of transiently identifiable, stereotypically arranged cell internalization sites; iii) immigration of predominantly post-mitotic ganglion cells; and iv) restriction of tangentially oriented cell proliferation to the apical cell layer. However, in the second phase, the formation of a central invagination in each hemi-neuromere is accompanied by the differentiation of apical neural stem cells. The latter grow in size, show high mitotic activity and an asymmetrical division mode. A marked increase of ganglion cell numbers follows their differentiation. Directly basal to the neural stem cells, an additional type of intermediate neural precursor is found.

**Conclusions:**

Embryonic neurogenesis of *Pseudopallene* sp. combines features of central nervous system development that have been hitherto described separately in different arthropod taxa. The two-phase character of pycnogonid neurogenesis calls for a thorough reinvestigation of other non-model arthropods over the entire course of neurogenesis. With the currently available data, a common origin of pycnogonid neural stem cells and tetraconate neuroblasts remains unresolved. To acknowledge this, we present two possible scenarios on the evolution of arthropod neurogenesis, whereby Myriapoda play a key role in the resolution of this issue.

## Background

Nervous system development and adult neuroanatomy of arthropods provide ‘a wealth of valuable characters that can be used for phylogenetic inferences’ [1]. In fact, the nervous system has been considered ‘a particularly suitable organ in which to search for characters to reconstruct evolutionary relationships between the major arthropod groups’ [2]. This is due to the nervous system’s conserved basic architecture on the one hand, and on the other its lineage-specific structural diversity, which facilitates the specific comparison and possible homologization of neural structures and their respective sub-parts [3]. The basic architecture of the arthropod central nervous system is most evident during embryonic development. It is formed via segmental units of differentiating neural tissue, the neuromeres, which generate a rope-ladder-like axonal scaffold, comprising intra-segmental transverse commissural pathways and inter-segmental connectives [3-15]. Over the years, comparison of various features of adult neuroarchitecture and aspects of nervous system development have led to the formulation of scenarios on nervous system evolution and the proposition of different hypotheses on arthropod phylogeny [1,16-21]. Recently, first ‘neural cladistics’ have been performed, but are limited to adult neuroanatomical characters [22,23].

**Figure 1:**
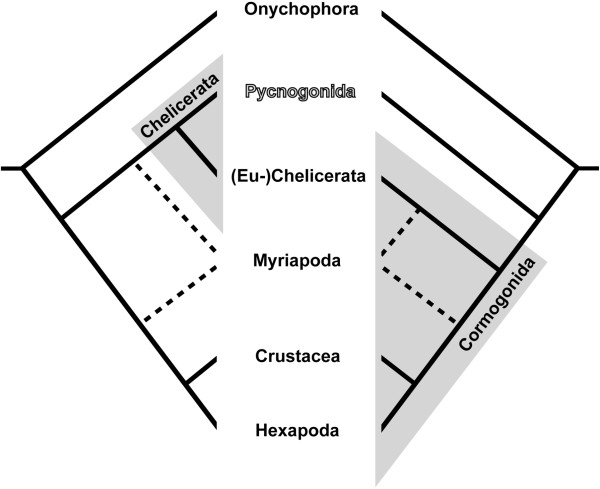
**Two hypotheses on the phylogenetic position of Pycnogonida within Arthropoda.** Left phylogram: Traditional placement of pycnogonids as sister group to Euchelicerata, both taxa forming the Chelicerata. Right phylogram: Alternative placement of pycnogonids as sister group to all remaining arthropod lineages, the latter representing the Cormogonida. Note the unresolved position of Myriapoda.

The developmental processes of early neurogenesis show distinct features in the major arthropod groups. In Hexapoda and at least some crustaceans (malacostracans and branchiopods), neurogenesis is coupled to a type of neural stem cell (NSC), the neuroblast (NB) [24-37]. NBs are comparably large and divide repeatedly in asymmetrical fashion, thereby ‘self-renewing’ and ‘budding off’ a smaller daughter cell – the ganglion mother cell (GMC) – into the interior of the embryo [38,39]. In turn, GMCs represent a neural precursor type that typically undergoes one terminal division, giving rise to immature post-mitotic neurons and/or glial cells [39,40], but see [41] for deviations. Recent work revealed detailed correspondences between NB cell lineages of a malacostracan crustacean (*Orchestia cavimana*) [35] compared to hexapods (e.g., *Drosophila melanogaster*) [42]. In addition to this striking similarity, correspondences in i) soma position, axon morphology and molecular marker expression of several pioneer neurons [29,35,43-45]; ii) ommatidium structure of the lateral eyes [46]; see also discussions in [47-49]; and iii) several additional features of adult neuroanatomy [18,19,21,23] indicate that hexapods and crustaceans form a monophyletic group (Figure 1), the Tetraconata [47]. This grouping is in good agreement with virtually all molecular analyses [50-55] and represents one of the uncontested nodes in the arthropod tree (though internal tetraconate relationships remain contentious) [56].

NBs are considered one of the apomorphies of Tetraconata, since no evidence for this NSC type has been uncovered during neurogenesis of Chelicerata and Myriapoda [14,57-64]. In these two taxa, mostly post-mitotic immature neurons/glial cells immigrate directly into the embryo in a hemi-segment-specific number of cell internalization sites (CISs), forming transient groups of flask-shaped cells. Cell proliferation occurs scattered in the neuroectoderm. However, in myriapods, cell proliferation appears to be linked to the CISs indicating that specialized neural precursors may be involved in neurogenesis [57,61,65], but the available data are still inconclusive. In line with these uncertainties, the phylogenetic placement of the myriapod lineage is insufficiently resolved (Figure 1). A chelicerate-myriapod-clade has been repeatedly suggested in molecular analyses [66-72] but stands opposed to the morphologically well-supported Mandibulata concept [23,73-75], which places myriapods as sister group to tetraconates. In this context, the similarities in early neurogenesis have been discussed as potential apomorphy of a chelicerate-myriapod-clade [61,62,76]. However, the increasingly strong support for the Mandibulata in recent molecular phylogenetic studies [50,51,55,77-80] has made the symplesiomorphic nature of the chelicerate-myriapod mode of neurogenesis more plausible. In accordance with this, this neurogenesis mode has been repeatedly suggested as an ancestral feature of the arthropod stem species [1,18-20,36,81].

Importantly, however, this conclusion still lacks new data on Pycnogonida, commonly known as sea spiders. They are an exclusively marine arthropod lineage with more than 1,300 described extant species [82], which has resisted confident phylogenetic placement since its first description [83]. The majority of recent molecular analyses tend to recover pycnogonids within Chelicerata, as sister group to all other chelicerate taxa (the latter henceforth called Euchelicerata in accordance with [84]) (Figure 1A) [51-53,55,78,80,85-87]. In spite of the comparatively low statistical support values for this grouping and a persisting paucity of apomorphies [83], it is currently better supported than the alternative hypothesis of a sister group relationship of pycnogonids and all remaining arthropods (Figure 1B) [88-92]. The few histological studies covering pycnogonid nervous system development report the presence of conspicuous large cells within so-called ‘ventral organs’ [93-96]. These ‘ventral organs’ represent segmentally arranged and bilaterally paired cell thickenings of the ventral neuroectoderm that are confluent with the basally underlying ganglion anlagen. The large cells are assumed to have high proliferation activity and have been interpreted almost unanimously as NSCs that produce ganglion cell material [93,94,96]. In one study [96], it is even claimed that pycnogonid neurogenesis begins with the differentiation of the large NSCs, which then generate radial columns of ganglion cells by repeated radial divisions. Considering our current scenarios on the evolution of arthropod neurogenesis, the description of presumptive NSCs in pycnogonids is highly intriguing. If their presence were to be confirmed, tetraconates would no longer be the only arthropod lineage being characterized by such specialized neural precursors. This might, accordingly, challenge the validity of NSCs as tetraconate apomorphy and call for a critical reassessment of current ideas on the evolution of arthropod neurogenesis.

This unresolved situation led us to study pycnogonid nervous system development. *Pseudopallene* sp., a pycnogonid representative of the Callipallenidae, was chosen for the investigations, its embryonic and post-embryonic development having been recently described [97,98]. In contrast to many other pycnogonid taxa, Callipallenidae do not hatch as free-living protonymphon larvae that bear a proboscis and just three pairs of limbs (chelifores plus palpal and ovigeral larval limbs) [99-102]; instead, they show a more pronounced embryonization of development [97,103-106]. This facilitates investigation of their development up to more advanced stages because embryos and early larvae are carried by the males throughout embryonic as well as early post-embryonic development and thus remain easily accessible. We applied a combination of fluorescent histochemical staining and immunolabelling coupled to confocal laser-scanning microscopy and computer-aided 3D analysis as well as classical histology to shed light on the neurogenic processes in pycnogonids at cellular level. We reveal two different modes of neurogenesis in *Pseudopallene* sp., occurring in two sequential phases of embryonic development. Neurogenesis is initially characterized by immigration of groups of flask-shaped and mostly post-mitotic cells from the neuroectoderm. In a subsequent phase, larger NSCs differentiate, which are then involved in the production of a notable amount of future ganglion cells. The obtained data for *Pseudopallene* sp. are compared to other pycnogonid species. Subsequently, they are critically evaluated in light of the currently best-supported hypothesis on arthropod phylogeny. Based on this, we discuss two feasible scenarios on the evolution of arthropod neurogenesis.

## Methods

### Specimen collection and fixation

Details on the collection of *Pseudopallene* sp. are given in Brenneis et al. [97]. Fixation of developmental stages was carried out at ambient temperature. For all fluorescence stainings, embryos were fixed in PFA/SW (16% formaldehyde in ddH_2_0 (methanol-free, Electron Microscopy Sciences, #15710) diluted 1:4 in filtered natural sea water). With the exception of a single batch of embryos that could be freshly fixed for 30 min in the laboratory in Berlin and processed directly afterwards, fixation was conducted either for 30–40 min with subsequent gradual transfer into absolute methanol for long-term storage, or over a prolonged time span (several days at ambient temperature plus some weeks at 4°C) with subsequent transfer into PBS (1.86 mM NaH_2_PO_4_, 8.41 mM Na_2_HPO_4_, 17.5 mM NaCl; pH 7.4) containing 0.1% NaN_3_. Storage in methanol was observed to result in shrinkage of the cytoplasmic compartment of the embryonic cells, especially in early morphogenesis stages, thus proving sub-optimal for analyses of cell shapes in the embryonic ectoderm. For histology, embryos were fixed in Bouin’s solution (15 parts saturated aqueous picric acid, 5 parts 37% formaldehyde (methanol-stabilized), 1 part glacial acetic acid) for 30–40 min, followed by repeated thorough washing and long-term storage in 70% ethanol.

### Obtainment of developmental stages of additional pycnogonid representatives and spider species

Some embryos of *Callipallene* sp. (Pycnogonida, Callipallenidae) were obtained from a single 70% ethanol-preserved male from Eaglehawk Neck, Tasmania.

Freshly hatched larvae of *Stylopallene cheilorhynchus* Clark, 1963 (Pycnogonida, Callipallenidae) preserved in 70% ethanol were provided by David Staples (Museum Victoria, Melbourne, Australia).

A culture of *Pycnogonum litorale* (Ström, 1762) (Pycnogonida, Pycnogonidae) is being constantly kept in the laboratory in Berlin (see [107] for more details on husbandry). Embryos were fixed in PFA/PBS (4% formaldehyde (methanol-free) in PBS) for 30 min at room temperature and directly processed after fixation.

Early larvae of *Nymphon gracile* Leach, 1814 (Pycnogonida, Nymphonidae) were collected in June 2006 during low tide at the rocky seashore of the ‘Station Biologique de Roscoff’, Bretagne, France, fixed in FA/PBS (3.7% formaldehyde (methanol-stabilized) in PBS) for 30 min at room temperature and transferred into absolute methanol for long-term storage.

Fixed embryonic stages of the two spiders *Cupiennius salei* (Keyserling, 1877) and *Parasteatoda tepidariorum* (Koch, 1841) were provided by Carsten Wolff, Humboldt-Universität zu Berlin (see [108,109] for fixation procedures).

### Egg membrane removal, specimen dissection and fluorescent staining procedures

Embryonic samples were manually freed from the surrounding layers of elastic matrix (produced by the males to glue the eggs into batches) and from the egg membrane, using ground dissection needles or electrochemically etched tungsten tips in combination with sharpened watchmaker forceps (Dumont 5). With the same tools, embryonic yolk was carefully removed from the germ bands, allowing flat preparations for subsequent analyses.

Labelling of F-actin with phallotoxins yielded positive results only for the freshly fixed *Pseudopallene* embryos, avoidance of extended storage periods appearing pivotal for this labelling approach. Samples were incubated in TRITC-coupled phalloidin solution (Sigma, #P1951, 50 μg/mL in PBS) for 90 min at room temperature. For immunohistochemistry, samples were initially washed in several changes of PBTx (0.3% Triton X-100, 0.5% bovine serum albumin, 1.5% DMSO (dimethylsulfoxide) in PBS) for ≥2 h and then blocked for ≥1 h in PBTx + N (5% Normal Goat Serum (Dako, #X0907) in PBTx) prior to antibody exposure. Primary and secondary antibodies (see below) were diluted in PBTx + N, incubation times lasted at minimum overnight, being sometimes extended up to 72 h. Each antibody incubation was followed by extensive washing in PBTx on a horizontal shaker (NeoLab® DOS-20S, 55–70 rpm) for at least 4 h at RT with several changes of the buffer. Omission of primary antibodies resulted in complete signal loss. Larvae of *S. cheilorhynchus* were incubated overnight at 4°C in the lipophilic marker FM 1-43FX (Invitrogen Molecular Probes®, #F35355, 5 μg/mL in ddH_2_O). Nuclear counterstaining was performed with Hoechst (H33342, Invitrogen Molecular Probes®, #H1399, 1 μg/mL in PBS) following the preceding labelling procedures. Hoechst incubation lasted at least 1 h and was occasionally extended overnight at 4°C. After final washing, samples were transferred into Vectashield® Mounting Medium (Vector Laboratories, Inc.) and cleared overnight at 4°C. For mounting, small pieces of plasticine were fixed to the corners of cover slips, acting as spacers that prevent squeezing of objects.

### Applied antibodies and antisera

#### Acetylated and tyrosinated alpha-tubulin

Heterodimers of alpha-tubulin and beta-tubulin are the basic components of microtubules, which represent an important part of the cellular cytoskeleton. They are subject to post-translational modifications, two of them being acetylation/deacetylation and detyrosination/tyrosination [110]. Acetylation is a common phenomenon and found in many cell types, being often encountered in stable microtubule assemblies [111]. Microtubule acetylation is non-uniformly distributed in neurons [110] and recent evidence suggests a critical role of acetylation of alpha-tubulin in neuronal migration, dendrite projections and arborizations [112]. Several studies have already used acetylated alpha-tubulin labelling to follow the establishment of the major axonal pathways during the development of different arthropods [5,7,12,13,15,113], including pycnogonids [114,115]. In pycnogonids, the cytoskeleton of all embryonic cells is intensely labelled by a monoclonal antibody against acetylated alpha-tubulin (mouse mab 6–11 B-1, Sigma, #T6793, dilution 1:100), which allows assessment of cell shapes in the ectoderm during development.

The gene-encoded protein of alpha-tubulin is characterized by a C-terminal tyrosine (= tyrosinated alpha-tubulin), which can be reversibly removed via detyrosination [111]. In interphase cells, microtubules enriched in tyrosinated alpha-tubulin have been indicated to be more dynamic than microtubules rich in detyrosinated tubulin [116]. The tyrosinated sub-class is therefore expected in regions of dynamic change of the cytoplasm, for instance in the growth cones of neurons [110]. As alternative to acetylated alpha-tubulin labelling, a monoclonal antibody against tyrosinated alpha-tubulin (mouse mab TUB-1A2, Sigma, #T9028, dilution 1:500) was applied to assess ectodermal cell shapes, which are subject to dynamic changes due to cell movements relating, amongst others, to neurogenesis. During mitosis, tyrosinated tubulin shows a similar distribution to total tubulin [117] and allows visualization of the spindle apparatus, which holds as well for acetylated alpha-tubulin (GB, personal observation).

#### Phosphorylated histone H3 (PH3)

An IgG fraction of a rabbit antiserum against phosphorylated histone H3 [pSer^10^] (Sigma, #H0412, dilution 1:200) was used to reveal the location of cells undergoing mitosis. In eukaryotic cells, histones represent a major component of the chromatin. Phosphorylation of histone H3 is positively correlated with mitotic chromosome condensation [118] and is in at least one site of the amino-terminal tail (serine 10) conserved throughout eukaryotes [119]. In *Pseudopallene* sp., the antiserum against the Ser^10^ phosphorylation site could be used to reliably visualize dividing cells in pro-, meta- and early anaphase. Mitoses in advanced anaphase or telophase were frequently only weakly labelled. Since PH3 labelling was always performed in combination with a nuclear counterstain, co-localization of PH3 and DNA and reliable identification of advanced mitosis stages amongst the surrounding ectodermal cells was possible.

Primary antibodies were targeted with appropriate fluorochrome-coupled secondary antibodies (α mouse IgG (H + L) AffiniPure Cy™3, goat mab, Jackson Immunoresearch/Dianova, #115-165-003, dilution 1:200; and *α* rabbit IgG (H + L) Alexa Fluor® 488, goat mab, Invitrogen Molecular Probes®, #A11038, dilution 1:400–1:500).

### Confocal laser-scanning microscopy (CLSM) and analysis of CLSM data

Image stacks were taken with a Leica DM IRE2 confocal laser-scanning microscope equipped with a Leica TCS SP2 AOBS laser-scan unit. Depending on the intended z-resolution, step sizes from 0.3 μm to 2.00 μm were chosen between successive scanning planes. Based on the emission characteristics of the applied fluorochromes, a combination of UV laser (405 nm wavelength → Hoechst), argon laser (488 nm wavelength → Alexa Fluor® 488) and helium-neon laser (543 nm → Cy™3, TRITC, FM 1-43FX) was selected for the recordings.

Analyses of the CLSM data were performed with the 3D reconstruction program ‘Imaris’ (Bitplane AG, Switzerland, version 7.0.0). Within the ‘Surpass mode’ of this program, 3D volumes are generated from the recorded image stacks. A volume can be turned and rotated in every spatial dimension, zoomed in and out and be modified by a number of additional tools.

Counts of the overall cell numbers within hemi-neuromeres were performed with the ‘Spots’ tool in conjunction with an ‘Ortho-slicer’. The combination of both tools allows manual marking of structures (here nuclei) coupled to an automatic ‘blind’ counting of applied spots. Due to ambiguities regarding the exact extensions of the ventral neuroectoderm in embryonic stage 2–3, and in part still in embryonic stage 3, cell counts in these early stages were performed to represent maximal estimators, i.e., cells at the borders of a hemi-neuromere (including the ventral midline region) were included rather than excluded from the count.

Nucleus measurements in the neuromere of walking leg segment 1 were performed with the ‘Measurement Points’ tool. Measurements were conducted independently in at least two embryos of the same developmental stage. Ten nuclei were measured in each hemi-neuromere, i.e., twenty nuclei per specimen. They were measured along their elongated axis, which in the great majority of cases was oriented in an approximately apico-basal direction. This analysis was performed to gain a general idea of ectodermal cell sizes during the course of development.

In order to depict arrangements of cells and cell groups within apical or basal cell layers of curved neuroectodermal regions more precisely, several ‘Oblique Slicers’ were combined. They were orientated to follow the curvature of the targeted cell layer, creating a 3D-curved virtual section plane. An image taken from such a curved plane represents a 2D projection of the cell layers instead of a true planar section. As a consequence, spatial arrangements of sub-structures towards the periphery of the image appear slightly closer spaced than in reality. However, for the purposes of the analyses, the advantages of this approach clearly outweighed these slight distortions.

The ‘Extended section mode’ of Imaris allows simultaneous visualization of virtual transverse, horizontal and sagittal sections with individually definable thickness (via inclusion of a variable number of images). The ‘Blend’ option of Imaris renders scanned structures non-transparent and thus facilitates evaluation of surface and external shape of an object.

### Histology

Bouin-fixed embryos were embedded in the plastic resin Technovit 7100 (Kulzer Histo-Technik) following the manufacturer’s standard protocols. Semi-thin sections (1.5 μm) were cut with a Microm HM 355 microtome, stretched at 60°C on a heating plate and stained in a first step with methylene blue-azure II solution, followed by a counterstain in basic fuchsin solution. Sections were embedded in Roti®-Histokitt (Roth) under cover slips. Photographs of selected sections were taken with a Zeiss Axioskop 2 plus microscope equipped with a digital camera (Zeiss AxioCam HRc).

### Data presentation

Global contrast and brightness values of some of the images were adjusted using Adobe Photoshop CS3. Figures were compiled in Adobe Illustrator CS3. If not stated otherwise, anterior is i) to the top in all ventral and dorsal aspects and in horizontal sections and ii) to the left in lateral aspects and sagittal sections. In anterior or posterior aspects and transverse sections, dorsal is to the top.

## Results

### Notes on the applied terminology and general aspects of neurogenesis in *Pseudopallene* sp

Distinction of developmental stages of *Pseudopallene* sp. follows the system of embryonic stages (ESs) recently presented by Brenneis et al. [97].

For descriptions of nervous system development, the terminology suggested by Richter et al. [39] has been followed whenever applicable. In accordance with Whitington [120], the term ‘neurogenesis’ is here used in a restrictive manner, covering only the processes that lead to the generation of post-mitotic but still immature neurons and glial cells. Accordingly, all subsequent cell differentiation processes, such as axonogenesis, are discriminated and excluded from neurogenesis. In recent literature, the term ‘neural precursor’ (NP) has been used to designate different cell types during nervous system development [120,121]. Here, it is applied in line with Richter et al. [39] and Whitington [120], being restricted to neurogenesis and designating a progenitor cell that has already entered the neural pathway but is not yet post-mitotic. Hence, a NP divides at least once more, giving rise either to further NPs or to post-mitotic immature neurons and/or glial cells. The neutral term ‘ganglion cell’ (GC) has been chosen to accommodate post-mitotic neurons and glial cells in all differentiation stages, since unequivocal distinction especially between immature cells of both types was not possible with the applied techniques.

The general events of early neurogenesis in *Pseudopallene* sp. were found to be the same in all ventral neuromeres. The neuromeres of the palpal and ovigeral segments and of walking leg segments 1 and 2 develop early on during embryonic morphogenesis and form distinct ganglion anlagen in late stages. Only a weak antero-posterior developmental gradient is observable between these neuromeres, their segment primordia having been already pre-patterned in the germ band of ES 2 [97]. Only in ES 8 and later, first neurogenic processes posterior to the anlage of walking leg ganglion 2 are detectable, relating to the neuromere of walking leg segment 3. From ES 4 onwards, the palpal and ovigeral neuromeres are increasingly covered by the distal portions of the elongating and anteriorly shifting chelifore anlagen [97], impeding unhindered observation of these neuromeres in flat-preparations of germ bands. In contrast to this, the neuromeres of walking leg segments 1 and 2 remain freely visible for the major part of embryogenesis, being only in the latest stages (ES 9 and ES 10) covered by the medially extending walking leg anlagen [97].

### Early neurogenesis in the ventral neuroectoderm (ES 2 – ES 4)

#### Onset of cell immigration and formation of local cell internalization sites

**Figure 2:**
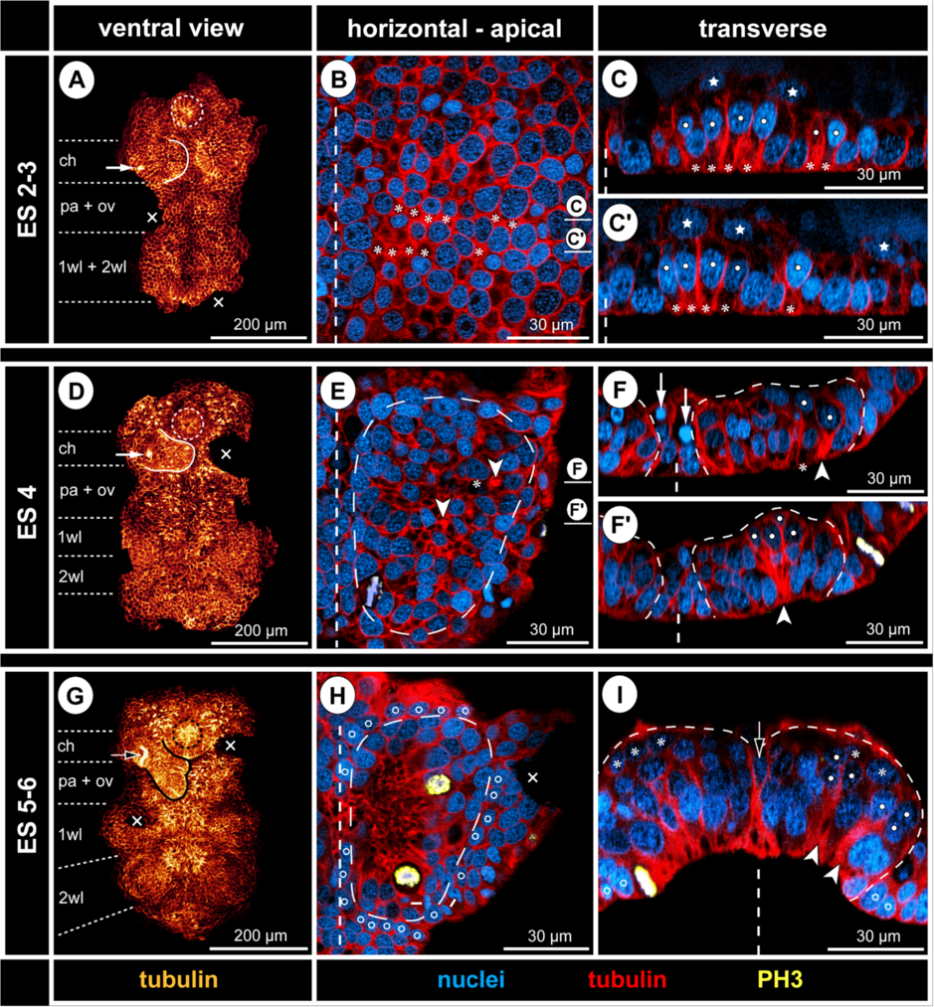
**Early neurogenesis in *Pseudopallene *sp. (late ES 2 – late ES 5).** Ventral overview of germ bands (tubulin) and optical sections through hemi-neuromeres of walking leg segment 1 (tubulin-, PH3- and nuclear labelling). Horizontal sections represent 2D projections of curved composite sections. Stippled circles mark stomodeum or pharynx. White arrows in **A** and **D** indicate immigrating cells related to spinning gland development. Black arrow in **G** marks spinning gland duct. Crosses indicate damaged germ band regions or virtually removed chelifore bud. Vertical dashed lines mark the VMR. **(A–C’)** Initiation of cell immigration, late ES 2. More intensely tubulin-labelled areas relate to constricting cell cortices (asterisks) of columnar cells with basally displaced nucleus (white spots). CISs are not yet recognizable. The VNE is underlain by scattered entodermal cells (stars). **(D–F’)** Formation of CISs, ES 4. Stippled outlines **(E–F’)** mark hemi-neuromere extensions. CISs have become defined in the VNE (tubulin-stained spots in **D**, selection marked by arrowheads in **E**). The nuclei lie in about three apico-basal levels, immigrating flask-shaped cells extending far basally (white spots in **F**’). Pycnotic bodies (white arrows in **F**) indicate occurrence of cell death. **(G–I)** Formation of central invagination and hemi-ganglion anlagen, late ES 5. Stippled outlines **(H,I)** mark hemi-neuromere extensions. CISs are still discernible in pre-cheliforal lobe and the palpal and ovigeral neuromeres **(G)**. Occasionally, cell arrangements reminiscent of CISs are found in the walking leg hemi-ganglion anlagen (white arrowheads and white spots in **I**). During mitosis, chromosomes and newly forming nuclei are encountered apically within the nucleus-free invagination **(H)** that is surrounded by smaller epidermal cells (open spots). The medio-lateral extension of the VMR has diminished, its remaining basal cells being often unpaired and wedged between the hemi-ganglion anlagen (open arrow in **I**). First GCs have detached basally (asterisks in **I**).

In early ES 2, the ventral ectoderm of the germ band is single-layered, consisting predominantly of columnar cells with apical nuclei that are elongated in apico-basal direction. It is underlain by a single layer of flattened and loosely distributed entodermal cells, which persists also during subsequent embryonic development (Figure 2C,C’).

In late ES 2, cell immigration is initiated in the medial ectoderm region, marking the onset of an apico-basally thickening of the ventral neuroectoderm (VNE). The first sign of cell immigration from the apical epithelium into the interior of the embryo is a basally directed displacement of the nuclei of some of the columnar ectodermal cells (Figure 2C,C’). The apical portion of these cells narrows slightly during and after nucleus migration and tubulin labelling shows their cortex more intensely stained than in the surrounding cells (Figure 2B–C’). Immigrating cells are found singly within the VNE, in small clusters or in row-like arrangements. Yet, neither a bilaterally symmetrical pattern, nor a repeated spatial arrangement along the antero-posterior axis can be detected at this stage.

**Figure 3:**
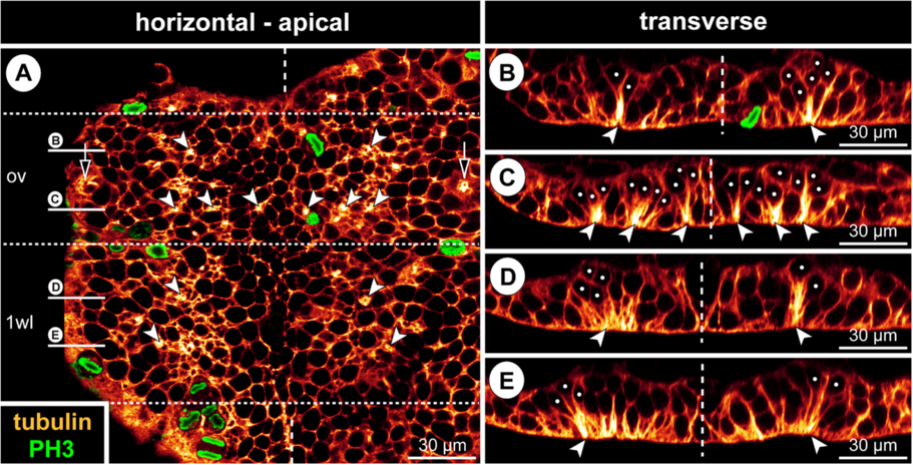
**Bilaterally symmetrical arrangement of CISs in the VNE of *Pseudopallene *sp. (ES 4).** Optical sections of tubulin- and PH3-labelled embryos. **(A)** 2D projection of a curved composite section. Vertical dashed lines indicate the VMR. White spots mark the basal nucleus-containing portions of flask-shaped cells in CISs. Arrowheads mark the apically converging cell extensions of flask-shaped cells in CISs. VNE of ovigeral and walking leg 1 segments, presumptive segment borders marked by horizontal dashed lines. Note differences in the distinctness of the tubulin-labelled spots of the CISs, the apically converging cell extensions of flask-shaped cells being in some cases placed less condensed. A largely bilaterally symmetrical pattern of CISs is recognizable. Note apical mitoses. Arrows indicate a conspicuous site of cell immigration lateral to each ovigeral hemi-neuromere, relating either to peripheral nervous system or gland development. **(B)** Transverse section through anterior portion of ovigeral neuromere. The two contra-lateral CISs of a bilaterally symmetrical pair do not always comprise a similar number of immigrating cells. **(C)** Transverse section through posterior portion of ovigeral neuromere. The transversally arranged CISs are closely spaced with only one or maximally two apical cells in between. **(D,E)** Transverse sections through anterior and posterior portion of walking leg neuromere 1, respectively. While the morphologically left CISs are well-defined, their contra-lateral counterparts are less distinct, being part of a wider region of cells with basally displaced nucleus (compare also to **A**). Presumably, the morphologically right hemi-neuromere would have predated the left one in the formation of the central invagination characteristic of ES 5.

**Figure 4:**
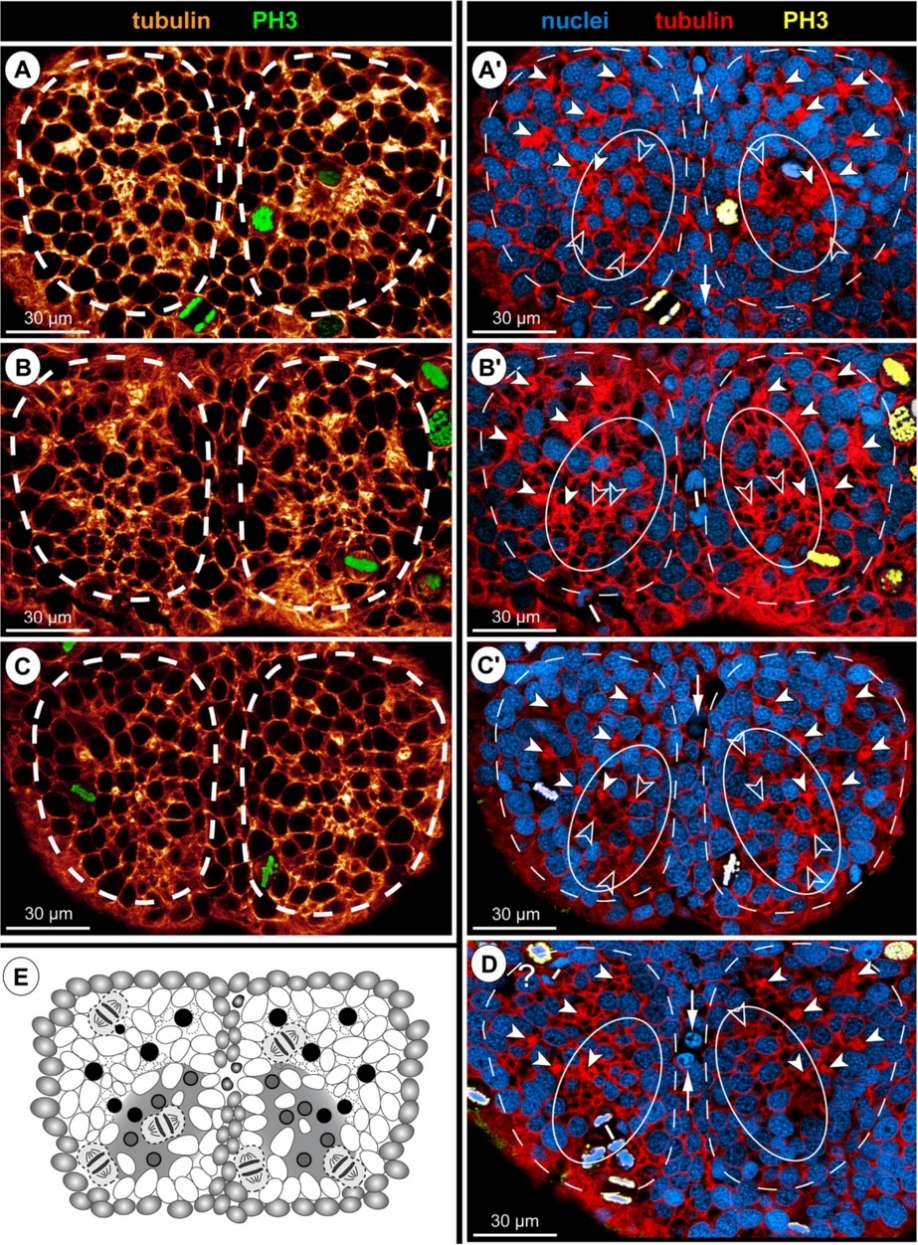
**Stereotyped CIS arrangement in walking leg neuromere 1 of *Pseudopallene *sp. (ES 4). (A,A’; B,B’; C,C’; D)** Apical horizontal sections (2D projections of curved composite sections) of four different tubulin- and PH3-labelled embryos, in part shown with nuclear counterstain. Images lacking nuclear counterstain **(A–C)** correspond to the ones shown to their respective right **(A’–C’)**. Dashed outlines indicate the extensions of the hemi-neuromeres. Solid arrowheads mark the six CISs that could be reliably identified in the great majority of specimens. Open arrowheads indicate less consistently encountered CISs in the postero-medial portion of the hemi-neuromeres. Ellipses highlight the region in which the central invagination begins to form. Arrows mark pycnotic bodies along the VMR. Note apical and in part clearly tangentially oriented divisions. The question mark in **(D)** indicates a region in which a typical antero-lateral CIS could not be securely identified, presumably due to transient rearrangements resulting from the nearby apical mitoses. **(E)** Scheme of apical horizontal view of walking leg neuromere 1 in ES 4. Black spots indicate reliably identified CISs, grey spots the more inconsistently identified CISs in the postero-medial region in which the central invagination (greyish area in the background) begins to form. Ectodermal cells with epidermal fate surrounding the hemi-neuromeres are depicted in grey; the cells in the VMR are shown slightly smaller. Small dark grey dots in the latter represent pycnotic bodies.

**Figure 5:**
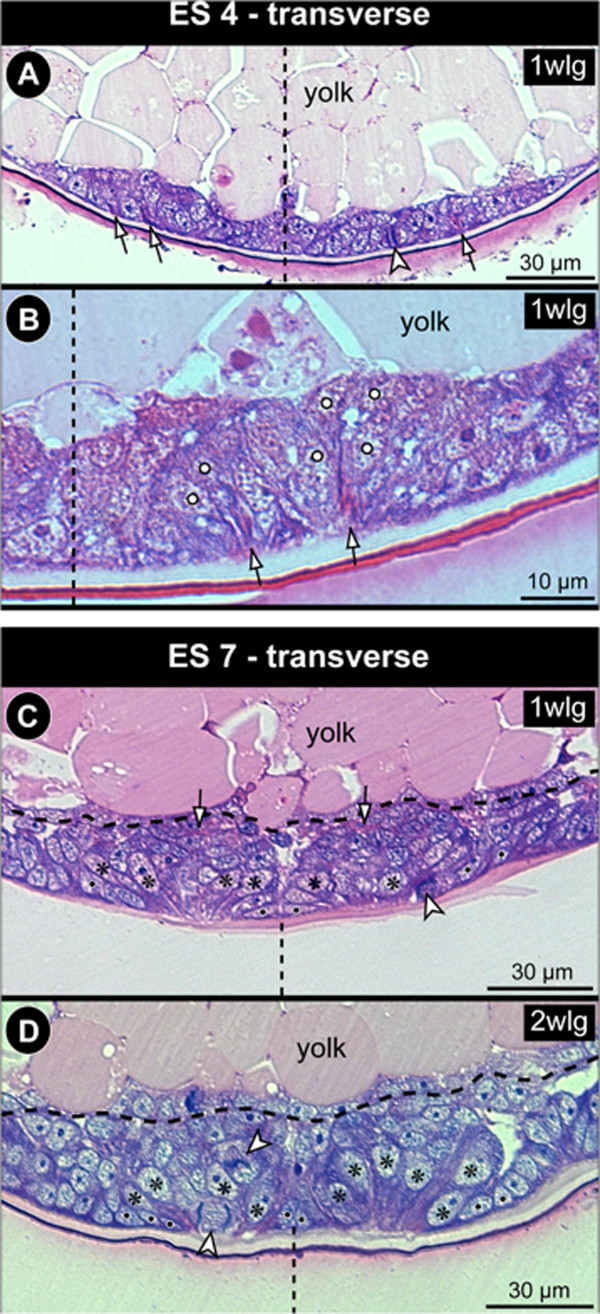
**CISs in the VNE and enlarged NSCs in *Pseudopallene *sp. (ES 4 and ES 7).** Transverse histological sections through ES 4 **(A,B)** and ES 7 **(C,D)**. The VMR is indicated by dashed vertical lines. Black spots mark small prospective epidermal cells. **(A)** Anterior portion of walking leg neuromere 1. The apically converging cell processes of three CISs are marked in the VNE (arrows). An apical tangential mitosis in anaphase is visible (arrowhead). **(B)** Posterior portion of right hemi-neuromere of walking leg segment 1. Apically converging cell extensions of two CISs are marked in the VNE (arrows). Note that the basally displaced nuclei (white spots) of the flask-shaped cells are not fully visible in this section, due to the immigrating cells’ oblique orientation. **(C)** Anterior portion of walking leg ganglion anlage 1. Note the spindle shape and less intensely stained nuclei of the NSCs (asterisks). One NSC is found in division (arrowhead). The ganglionic neuropil has started to differentiate basally (arrowheads). **(D)** Posterior portion of walking leg ganglion anlage 2. Note the larger nuclei and more voluminous cytoplasmic compartment of the spindle-shaped NSCs (asterisks). Two divisions are marked (arrowheads). The large apical one relates clearly to a NSC, whereas the nature of the sub-apical mitosis was not unambiguously resolvable in the section series.

**Figure 6:**
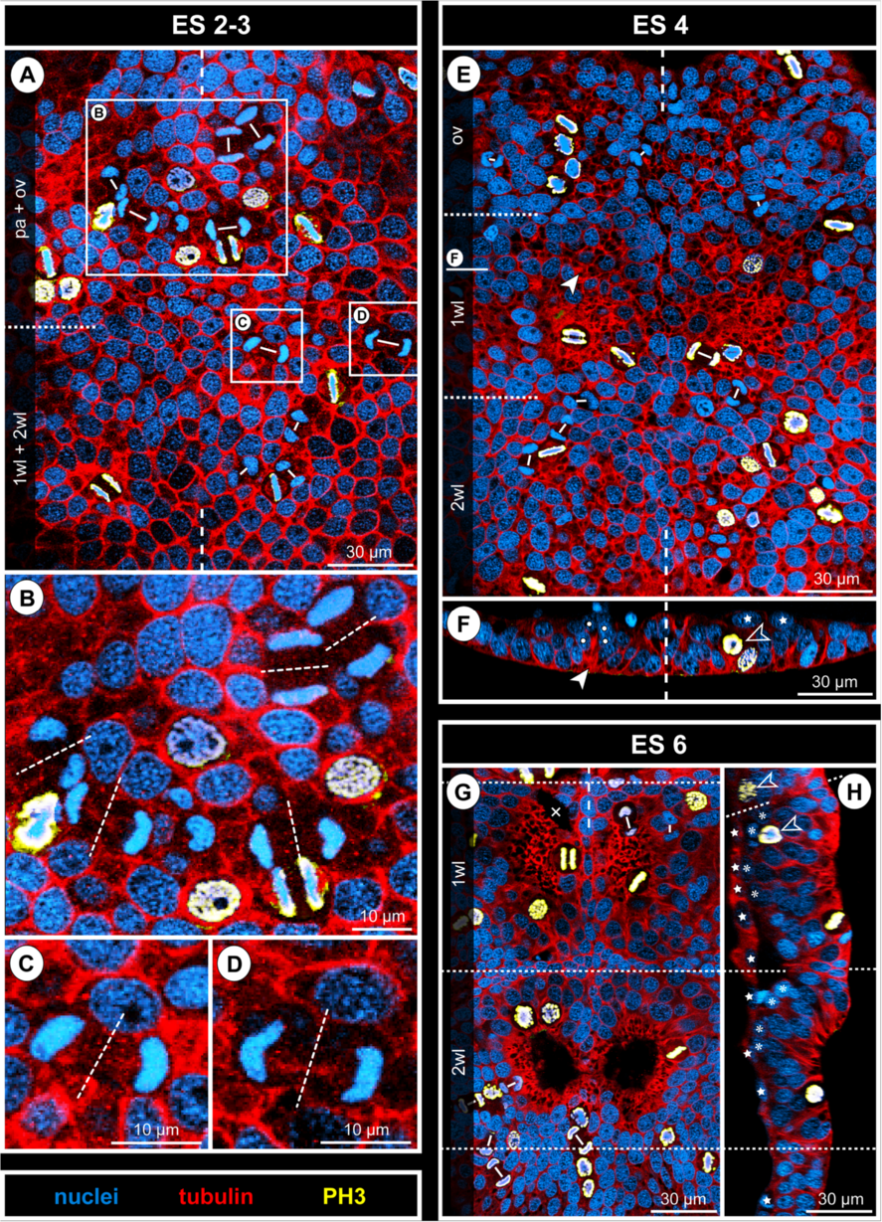
**Mitotic activity in the VNE and early hemi-ganglion anlagen of *Pseudopallene *sp. (ES 2 – ES 6).** Optical sections of tubulin- and PH3-labelled embryos with nuclear counterstain. Apical horizontal sections **(A,E,G)** represent 2D projections of curved composite sections. Dashed vertical lines indicate the ventral midline region. Dashed horizontal lines mark presumptive segment borders. Stars **(F,H)** mark embryonic entodermal cells. **(A–D)** Late ES 2. Rectangles in **A** highlight the magnified regions shown in **B–D**. Mitoses are exclusively found apically in the VNE. Note the tangential orientation of cell divisions, clearly recognizable during meta-, ana- and telophase of mitosis. Small white lines in **A** connect the forming nuclei of two sister cells during telophase. There is no preferred orientation of the divisions within the horizontal plane, all divisions being symmetrical (delicate stippled lines in **B–D** indicate the plane of cytokinesis). **(E,F)** ES 4. Mitoses are still encountered in the apical cell layer of the neuromeres. The formation of the central invagination of the postero-medial portion of walking leg neuromere 1 is already advanced. White solid arrowhead marks one of the CISs in the anterior portion of this neuromere. White spots in the corresponding transverse section **(F)** highlight the nuclei of flask-shaped cells in this CIS. In its contra-lateral counterpart, an exceptional division of a cell with slightly basally displaced nucleus is found (open arrowhead). **(G,H)** ES 6. Mitoses occur in the centre and at the margins of the paired invaginations of walking leg neuromeres 1 and 2. Note the tangential orientation of the mitoses in meta-, ana- and telophase. The cross **(G)** marks a damaged region. In the sagittal section **(H)**, two of the scattered basal NPs in mitosis are indicated (open arrowheads). Asterisks mark detached basal GCs.

From ES 3 to ES 4, basal displacement of nuclei is observed in an increasing number of cells in the VNE. For this reason, the latter assumes a distinctly stratified structure, the cell nuclei being arranged at first in two and later in three apico-basal levels (Figures 2F,F’ and 3B–E). The denser cell packing not only allows more reliable demarcation of the VNE from the more lateral ectodermal regions, but also morphological delimitation of the segmental neuromeres along the antero-posterior axis. Up to this point, none of the immigrating cells seems to have detached from the apical ectoderm. In an increasing number, the apical nuclei-free cell portion has constricted to a slender, intensely tubulin-stained process, resulting in a flask-like cell shape (Figures 2F,F’ and 3B–E). Immigrating flask-shaped cells start to be clustered in several small groups per hemi-neuromere (Figures 2F,F’ and 3B–E). In tubulin-labelled embryos, such localized CISs are detectable as more intensely stained spots among the cortical cytoskeleton of surrounding apical VNE cells (Figures 2D,E, 3A and 4). Also in histological sections, some CISs can be discerned in the closely packed VNE (Figure 5A,B), but their reliable identification on single sections is hampered by different oblique orientations of the immigrating cells of each CIS. Notably, the degree of convergence of the apical cell processes within a CIS varies between different embryos and within the same specimen. Due to this, the tubulin-positive spots are in some cases small and distinct, in others bigger and more diffuse (Figures 3A and 4). CISs are positioned in close proximity to each other, being often separated only by a single cell with apical nucleus (Figures 3A,C, 4, 5A and 6E). Where clearly assessable, the CISs of ES 4 were observed to include four to six, rarely also more flask-shaped cells. CISs are often surrounded by cells whose nuclei are basally displaced and appear to be still in the initial processes of cell immigration (Figures 2F and 6F). With further development, these cells will most likely be included in the CIS they adjoin. Hence, it is unlikely that CISs are characterized by fixed cell numbers. Rather, numbers seem to vary due to ongoing recruitment of additional cells from the apical layer.

**Figure 7:**
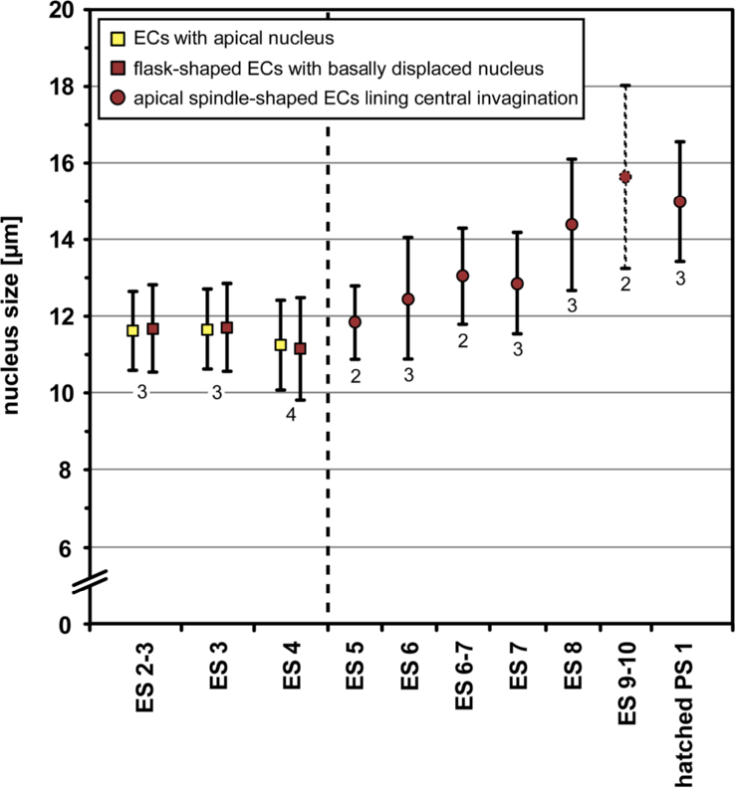
**Nucleus measurements in walking leg neuromere 1 of *Pseudopallene *sp. (late ES 2 – PS 1).** The ellipsoid nuclei of the VNE have been measured along their elongated axis. Arithmetic mean is shown, bars represent standard deviation. Small numbers below the values give the number of specimens analysed. Up to the formation of the central invagination (indicated by dashed vertical line), flask-shaped immigrating cells with basally displaced nucleus (dark red squares) have been measured in comparison to cells with apical nucleus (yellow squares). From the formation of the invagination to the hatching of the first post-embryonic stage, the enlarging spindle-shaped cells lining the invagination were measured (dark red circles). The values obtained for late embryonic stages (ES 9–10, stipples outlines) represent most likely an overestimation, since during this developmental period the cells are considerably compressed and distorted due to the pressure exerted by the covering walking leg anlagen [97].

In the ectodermal cells of these early stages, the nucleus takes up the major part of the overall cell volume. Therefore, measurement of nucleus size was considered sufficient to assess differences in ectodermal cell sizes. In embryos from late ES 2 to ES 4, measurements in the VNE of walking leg segment 1 do not indicate distinct differences in nucleus size between the apically remaining cells (Figure 7, yellow squares) and the immigrating flask-shaped cells (Figure 7, red squares). The mean nucleus extension (measured along the elongated apico-basal axis) ranges between 11 and 12 μm in both cell categories. If any size change occurs over this developmental phase, it is a slight decrease in nucleus extension in both cell categories from ES 3 to ES 4 (Figure 7).

#### CIS pattern in walking leg neuromere 1 of ES 4

The neuromere of walking leg segment 1 was considered the most promising candidate for analysis of CIS patterns, owing to its easy accessibility and a comparably low extent of morphogenetic rearrangements in the course of embryogenesis (see above).

No specific temporal sequence in the formation of CISs was recognized, which includes a lack of distinguishable temporal ‘waves’ or ‘pulses’ in the emergence of CIS sub-sets. In ES 4, when CISs are most clearly defined, a largely bilaterally symmetrical arrangement in stereotyped positions is recognizable (Figure 4; see also Figure 3A for example of a largely bilaterally symmetrical arrangement in an ovigeral neuromere). Four CISs are positioned in the antero-lateral region of each hemi-neuromere in an arrangement resembling the corners of a trapezoid (Figure 4, anterior-most solid arrowheads). Posterior to them, two very closely adjoining CISs are found (Figure 4, posterior-most solid arrowheads). They are positioned at the lateral margin of a primordial central invagination that starts to develop during ES 4 due to ongoing immigration of neurogenic cells. This primordial invagination occurs at first only in the postero-medial regions of the hemi-neuromere, but expands to encompass its entire central region up to ES 5 (see below). Within it, four to five additional CISs in characteristic positions could be frequently – but not consistently – identified with the applied techniques (Figure 4, open arrowheads). Hence, the maximal number of CISs we could identify per walking leg hemi-neuromere 1 ranges from six to eleven in ES 4.

#### Cell divisions in the VNE during early neurogenesis

Throughout embryonic morphogenesis, cell proliferation can be observed in the VNE. However, we did not detect bilaterally symmetrical division patterns of identifiable cells at any time.

From ES 2 to ES 4, cell divisions are almost exclusively positioned apically in the VNE and do not show any traces of morphological asymmetry (Figure 6A–E; see also Figures 3A,B, 4 and 5A). Initially, they lie within the single-layered neuroepithelium. Following the initial stratification of the VNE, divisions are found in the apical-most nucleus level. The great majority of divisions have a tangential orientation, i.e., they occur in parallel to the surface of the VNE (Figures 3A, 5A and 6A–E). Accordingly, the two daughter cells lie, at least initially, side by side within the apical VNE layer. No indications of a preferred antero-posterior or medio-lateral direction of divisions are detectable. Occasional deviations from a perfect tangential orientation of meta- and anaphase profiles occur, especially in ES 4, when the regular arrangement of cells with apical nuclei has begun to disintegrate owing to continuing cell immigration around the CISs. However, the oblique inclination of such divisions is almost always closer to a tangential than to a radial orientation and is likely to represent a phenomenon related to the adjacent cell movements instead of an intrinsic change of the division mode. Divisions occur regularly but not exclusively in the direct vicinity of CISs (Figures 3A,B, 4 and 6E,F). Only very rarely do divisions take place in a more basal position in the VNE and, merely in two instances, a cell division was found among the flask-shaped cells of CISs (Figure 6F) which otherwise completely lacked mitotic activity. Hence, up to ES 4, the vast majority of immigrating cells in the VNE appear to be post-mitotic immature ganglion cells (GCs).

### Neurogenesis in the ventral neuromeres of advanced embryonic stages (ES 5 – ES 10)

#### Formation of hemi-ganglion anlagen with apical segmental invaginations

**Figure 8:**
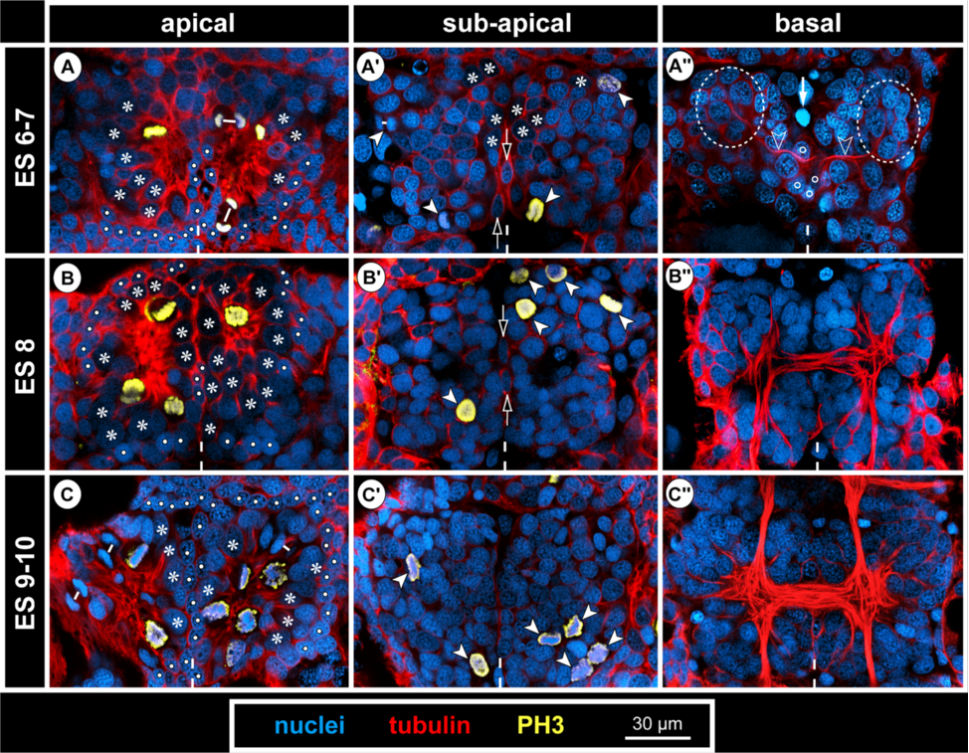
**Structure and mitoses in advanced walking leg neuromere 1 of *Pseudopallene *sp. (late ES 6 – ES 10) – part I.** Optical sections of tubulin- and PH3-labelled embryos with nuclear counterstain. Dashed vertical line at the bottom of each image indicates the VMR. Small white lines connect newly forming nuclei during telophase. **(A–C)** Apical horizontal sections. Large apical NSCs line the paired invagination (asterisks), some of them being in division. Prior to hatching **(C)** compression of the apical hemi-ganglion portion by the outgrowing limb anlagen leads to a squeezed appearance of the invaginations and cells. Smaller prospective epidermal cells (white spots) line the rim of the invaginations. **(A’–C’)** Sub-apical horizontal sections. Divisions of sub-apical INPs (arrowheads) are observable throughout this phase of embryonic neurogenesis, being predominantly located in the anterior and posterior regions of the hemi-ganglion anlagen. Open arrows mark unpaired median cells of the VMR (putative glial cells). **(A”–C”)** Basal horizontal sections. Axonogenesis starts in late ES 6 **(A”)**, the connectives being pioneered (not shown) and a commissural pathway (open arrowheads) being established by neurons located in the antero-lateral basal region of the hemi-ganglion anlage (stippled ovals). Medially, the growth cones have not yet crossed to the contra-lateral side and are in close contact to some of the basal cells of the VMR (open spots). Arrow marks a pycnotic body. During subsequent embryonic development **(B”,C”)** the slender transverse pathway differentiates into a compact undivided segmental commissure.

**Figure 9:**
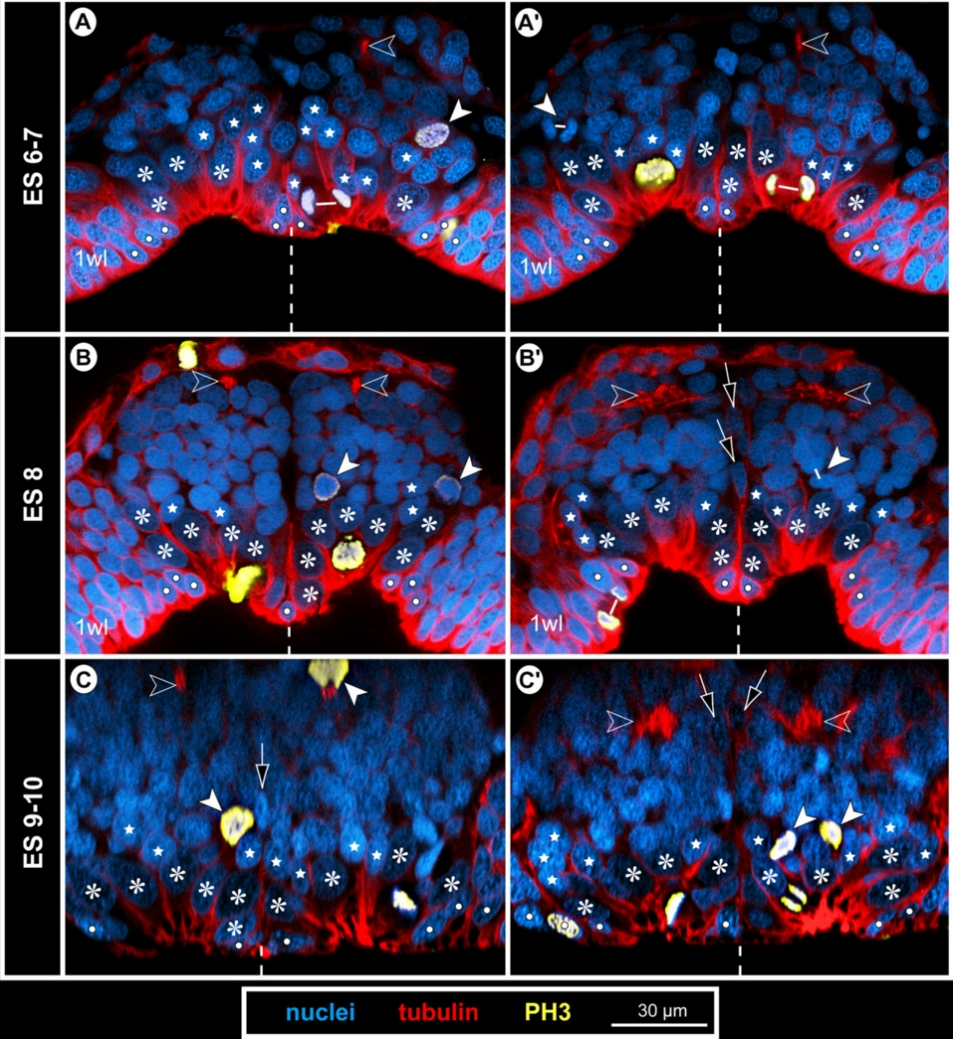
**Structure and mitoses in advanced walking leg neuromere 1 of *Pseudopallene *sp. (late ES 6 – ES 10) – part II.** Tubulin- and PH3-labelling with nuclear counterstain. Two transverse optical sections through the ganglion anlagen of late ES 6 **(A,A’)**, ES 8 **(B,B’)** and pre-hatching ES 10 **(C,C’)**, respectively. Dashed vertical line at the bottom of each image indicates the VMR. Small white lines connect newly forming nuclei during telophase. With ongoing development, the large apical NSCs (asterisks) are underlain by a growing number of GCs (including INPs). Stars label smaller flask-shaped cells that are in the process of immigration. Solid arrowheads mark sub-apical INPs in division. Open arrowheads mark developing neuropil and axonal pathways at the basal side of the ganglion anlagen. Arrows indicate flattened median cells of the VMR (presumably glial cells). The upper solid arrowhead in **C** marks one of the few basal NPs in division.

**Figure 10:**
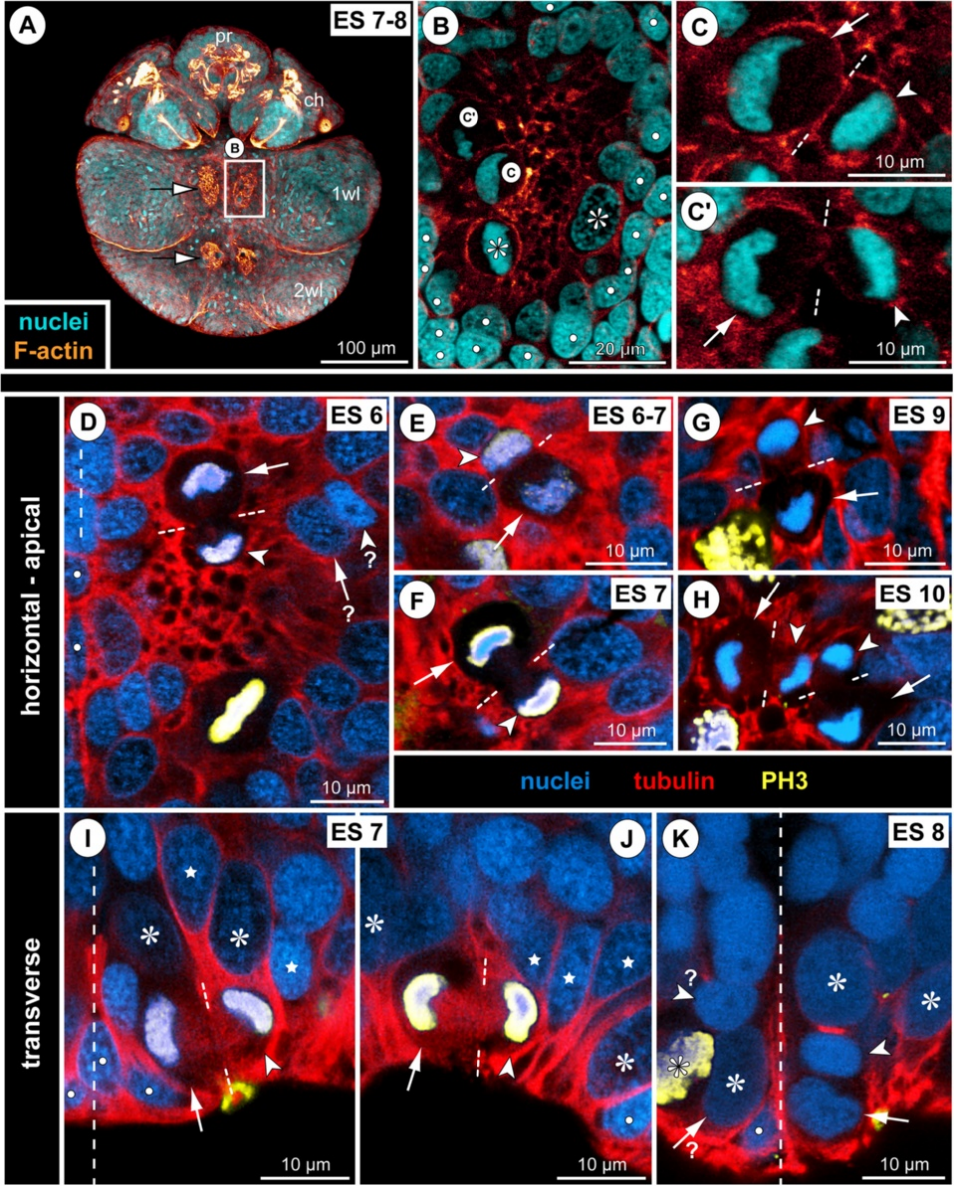
**Asymmetrical divisions of NSCs during advanced neurogenesis of *Pseudopallene *sp. (ES 6 – ES 10).** F-actin- **(A–C’)** and tubulin- and PH3-labelled embryos **(D–K)** with nuclear counterstain. Optical sections showing Imaris volume, except **(A)**. Oblique slicers have been aligned to show the asymmetry of forming sister cells as clearly as possible. Dashed vertical lines indicate VMR. White spots mark prospective epidermal cells surrounding the central invaginations. Asterisks mark NSCs that are not in division. White arrows mark larger sister cells of a NSC division (presumably self-renewing NSCs), arrowheads mark smaller sister cells of the same division. Note the asymmetrical positioning and different appearance of the newly forming nuclei. **(A)** Ventral view of entire embryo in late ES 7. Note F-actin-positive and largely nuclei-free paired invaginations in walking leg neuromeres 1 and 2 (black-framed arrows). In the proboscis and chelifore anlagen, intensely labelled structures represent differentiating muscles and parts of the spinning and chela glands. **(B)** Magnification of central invagination highlighted in **(A)**. Note absence of grid-like array of F-actin-positive spots within the invagination, indicating a lack of defined CISs at this stage. Four NSCs in division are apically visible, two of them in telophase (details in **C**,**C**’). **(D–H)** Horizontal/slightly oblique sections showing asymmetrical NSC divisions in hemi-ganglion anlagen of walking leg neuromeres. Question marks in **(D)** indicate possible sister cells of a previous NSC division. Note slight cell distortions in late embryonic stages **(G,H)** due to compression by the covering walking leg anlagen. **(I–K)** Transverse sections showing asymmetrical NSC divisions in hemi-ganglion anlagen of walking leg neuromeres. Stars mark smaller immigrating cells. Note radial arrangement of the forming nuclei at the rim of the invagination in ES 8 **(K)**. It remains unresolved whether the marked cells to the left (question marks) represent sister cells.

As a consequence of the ongoing basal displacement of nuclei in the remaining apical cells of the VNE, the surface in the centre of each hemi-neuromere becomes gradually nucleus-free (Figures 2H, 6G and 8A,B). Only cytoplasmic cell extensions remain in apical position and the basally displaced nuclei lie in a compact multi-layered mass that represents the hemi-ganglion anlage (Figures 2I and 6H). The nucleus-free apical region forms a shallow invagination that deepens considerably with ongoing development (Figures 2I, 6G,H and 9A–B’). In the walking leg neuromeres, this development begins already in late ES 4 (Figures 4 and 6E), being more pronounced from ES 5 onwards. In the palpal and ovigeral neuromeres, it takes place slightly later, during ES 6. Each central invagination of a walking leg segment is laterally flanked and increasingly compressed along the medio-lateral axis by the protruding limb bud (Figures 2I, 9A–B’ and 10A). Specific CISs are no longer reliably identifiable amongst the ubiquitous apical cell processes of the nuclei-free invaginations, neither by using tubulin labelling nor phalloidin staining (Figures 2G,H, 6G, 8A,B and Figure 9A,B, respectively). However, more closely attaching bundles of apical cell processes are sometimes still encountered in ES 5 and ES 6, and assignment of these processes to distinct groups of basally displaced nuclei was, in exceptions, possible (Figure 2I). In ES 5, the great majority of immigrating GCs are still attached to the apical surface, but first dissociated cells with apico-basally flattened nuclei start to be found at the basal side of the hemi-ganglion anlagen. Up to ES 6, a loose basal-most layer of detached cells has formed, being most pronounced at the anterior side of the growing hemi-ganglion anlagen (Figures 2I and 6H).

#### Apico-basal sub-structuring of the differentiating hemi-ganglion anlagen

In ES 5 and ES 6, cell proliferation is still found apically, close to the margins of each nuclei-free invagination and in its centre (Figures 2H,I and 6G,H). Apical cells in division are very conspicuous owing to their PH3-labelled chromatin and copious surrounding cytoplasm against the otherwise nuclei-free apical surface of the invagination (Figure 6G,H). In comparison to the preceding stages, a beginning enlargement of apical-most cells lining the invaginations is observed (Figure 7).

From late ES 6 onwards, transverse sections through ventral hemi-ganglion anlagen reveal three apico-basal cell regions, which become increasingly distinct with ongoing embryonic development.

i) Apical-most, large cells of spindle-shape with an ellipsoid nucleus line the central invagination. Intermingled between them are some smaller cells. The conspicuous large cells represent neural stem cells (NSCs, see below).

ii) Sub-apically, i.e., directly basal to the NSCs, a region comprising smaller cells is found, including intermediate neural precursors (INPs, see below).

iii) The basal-most layer in a hemi-ganglion anlage is for the most part formed by segregated immature GCs and already differentiated neurons and glial cells.

#### The apical neural stem cells (NSCs)

In nuclear counterstains, the ellipsoid nuclei of the spindle-shaped NSCs are less intensely labelled than those of the more lateral ectodermal cells and the more basal nuclei within the hemi-ganglion anlage (Figure 9). Similarly, they are only lightly stained by methylene blue-azur II in histological sections (Figure 5C,D). Measurements in the anlage of walking leg ganglion 1 demonstrate a distinct nucleus enlargement from ES 5 onwards (Figure 7). In ES 6, a mean value exceeding 12 μm is observed for the first time, which continues to increase to 15 μm until the end of embryonic development (Figure 7). Further, the cytoplasmic compartment of the NSCs seems to take up more space compared to the ectodermal cells in the VNE of the preceding stages (Figure 5). Accordingly, the measured increase of nucleus extensions even underestimates the enlargement of the total cell extensions.

The NSCs show notable proliferation activity (Figures 8A,B,C, 9 and 10), more than half of the labelled mitoses in the ganglion anlagen being encountered in the apical-most region. The NSCs divide asymmetrically (Figures 9A and 10). While the distinctly PH3-labelled earlier phases of mitosis (pro-, meta- and early anaphase) do not show any easily detectable deviations of a symmetrical cell division, morphological asymmetry becomes apparent in the weakly PH3-labelled late stages of mitosis, which are accompanied by the onset of cytokinesis (Figure 10). One of the forming daughter cells is larger, with a more voluminous cytoplasmic compartment, in the centre of which the chromatin of the newly forming nucleus has often a slightly curved shape (Figure 10C,C’). The other daughter cell is smaller, the chromatin of its newly forming nucleus being by comparison less curved (Figure 10C,C’) and often more intensely labelled. Apical asymmetrical NSC divisions occur throughout the remainder of embryonic development (Figure 10) and are still found in the freshly hatched larva (data not shown). Especially in ES 6 and ES 7, when the central invaginations are not yet very deep, the orientation of the NSC divisions is tangential or slightly oblique (Figures 5D, 8A, 9A,A’ and 10D,I,J). With increasing medio-lateral compression and deepening of the central invaginations, determination of the orientation of divisions becomes more difficult due to the compaction of the neuromeres (Figure 10G,H). Especially at the margins of the invaginations, strongly oblique and apparently radial orientations are detectable in late stages (Figure 10K, but see Discussion).

First cell counts of the NSCs in walking leg segment 1 have resulted in a range of 30 to 40 NSCs per hemi-ganglion anlage (counted in specimens ranging from late ES 6 to the hatching post-embryonic stage 1, n = 11). This range represents only a preliminary approximation, since individual nucleus sizes of the apical NSCs vary to a certain extent (Figure 7) and molecular markers that unambiguously discriminate NSCs from other cell types are still lacking. Nonetheless, the asymmetrical division mode and the more or less consistent range of larger NSCs over a long developmental period suggest that this cell type divides at least temporarily in stem cell fashion, leading to its self-renewal and a smaller daughter cell.

#### Sub-apical intermediate neural precursors (INPs)

A sub-apical layer of smaller cells with more intensely labelled nuclei lie basal to the apical NSCs (Figures 5C,D, 8A’,B’,C’ and 9). Some of these cells appear to be in the process of immigration, being flask-shaped with apically attached cell processes that extend between the apical NSCs (Figures 9 and 10I,J). From late ES 6 onwards, mitoses are observed in this sub-apical cell region, basal to the NSCs but still apical to the main mass of differentiating GCs (Figures 8A’,B’,C’ and 9). The mitosis profiles of the sub-apical cells are distinctly smaller compared to the large apical NSCs, and no signs of morphological asymmetry were detected. Accordingly, the sub-apical cell layer houses an additional type of intermediate neural precursor (INP). The sub-apical INP divisions are in particular located in the anterior and posterior portions of the hemi-neuromeres (Figure 8A’,B’,C’), prefiguring regions that during early post-embryonic development will retain the main connections between the apical region that accommodates the proliferating NSCs and the constantly growing basal hemi-ganglion anlagen proper (not shown). Given the tangential divisions of the apical NSCs and the ongoing presence of flask-shaped smaller cells in between the NSCs, it seems plausible that the sub-apical INPs represent smaller NSC daughter cells that have immigrated and then started to proliferate in a sub-apical position. However, with the available data this genealogical relationship cannot be proven directly.

#### Basal hemi-ganglion anlage with post-mitotic differentiating GCs and scattered basal INPs

The basal-most layer in a hemi-ganglion anlage comprises predominantly immature GCs and already differentiating neurons and glial cells (Figure 8A”,B”,C”). This layer is continuously gaining in size during ongoing development and constitutes the main portion of the hemi-ganglion anlagen in terms of volume and cell number in late embryonic stages (Figure 9). At its apical side, especially in the anterior and posterior regions of the hemi-ganglion anlage, it is confluent with the sub-apical INP cell layer. From ES 6 onwards, axonogenesis is detectable in the ventral hemi-ganglion anlagen (Figures 8A”,B”,C”,B and 9). Notably, few scattered mitoses occur in deep basal layers in the hemi-ganglion anlagen during the remainder of embryonic development (Figures 6H and 9C). Accordingly, not all basally located cells are already post-mitotic GCs. Instead, some of the basal cells still represent INPs that divide at least once before terminal differentiation. Although the exact ratio of basal post-mitotic GCs and INPs remains unresolved, the comparably low number of mitoses speaks in favour of a predominance of the GCs.

### Ventral midline region (VMR) and origin of the ventral epidermis

#### Stratification and compaction of the VMR during early neurogenesis

In ES 2 and early ES 3, the ventral midline region (VMR) is two to three cells wide, being enclosed on both sides by the VNE. The VMR of these early stages is generally characterized by the apical position of almost all its nuclei, even after immigration of cells has started in the more lateral VNE regions (Figure 2B–C’).

With development towards ES 4, the medio-lateral extensions of the VMR are gradually diminishing, its cells being wedged between the growing hemi-neuromeres (Figure 2E–F’). During this period, nuclei have become basally displaced in the VMR, leading to its stratified structure. If at all, VMR stratification remains only slightly less pronounced than in the laterally adjoining hemi-neuromeres (Figures 2F,F’, 3B–E, 5A,B and 6F). No median CISs are present in the VMR, but in some regions it is closely flanked by bilaterally paired, medial CISs from the adjacent hemi-neuromeres (Figure 3A,C). Mitoses with tangential orientation are observed apically (Figures 4B’ and 5A,B).

#### Cell death and further VMR compaction during formation of hemi-ganglion anlagen

**Figure 11:**
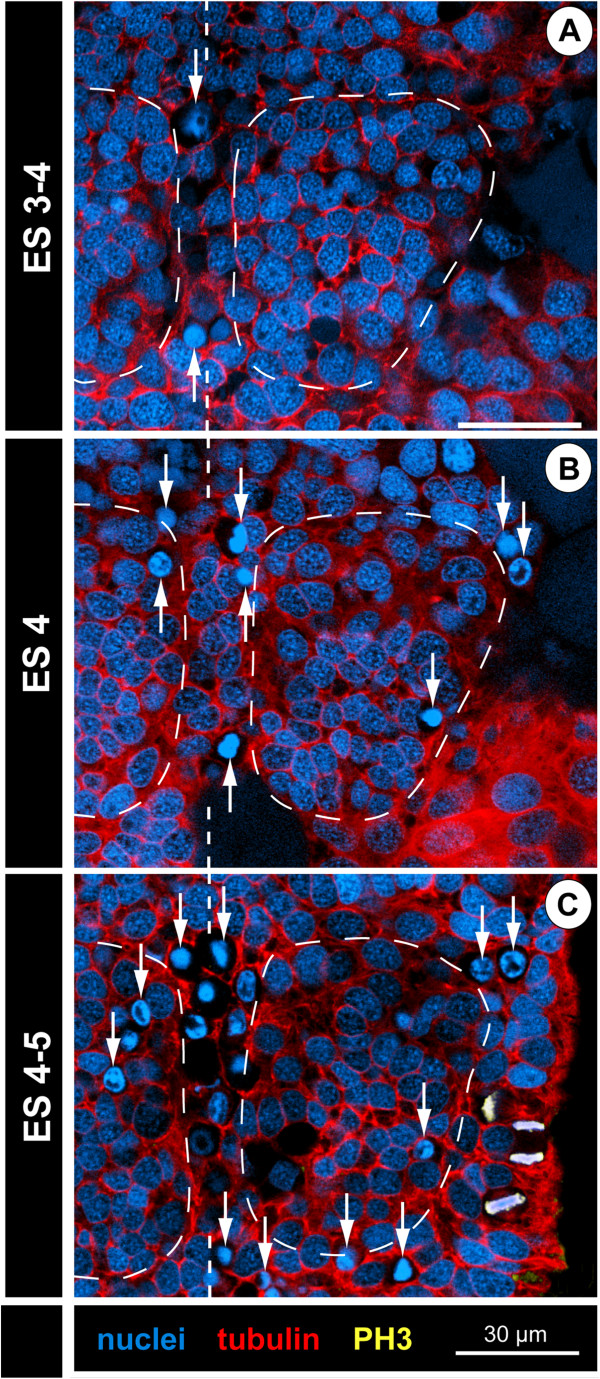
**Cell death in walking leg neuromere 1 of *Pseudopallene *sp. (late ES3 – late ES 4).** Horizontal optical sections of tubulin- and PH3-labelled embryos with nuclear counterstain. Sections are at sub-apical to basal level. Dashed vertical lines mark the VMR. Dashed outlines indicate extensions of the hemi-neuromeres. Note increase of pycnotic bodies (arrows) in the VMR from late ES 3 **(A)** over ES 4 **(B)** to late ES 4 **(C)**. To a lesser extent cell death occurs also within the developing hemi-neuromeres and at their lateral margins **(B,C)**. Virtually no cell divisions are found in the basally displaced cells of the hemi-neuromeres, the mitoses labelled in **(C)** are positioned apically in the downwards bent lateral ectoderm of this specimen.

From late ES 3 to late ES 4, nuclear labelling reveals an increasing number of small condensed bodies of heterogeneously stained chromatin amongst the cells of the medio-laterally compressed VMR (Figures 2F, 4A’ ,C’ ,D and 11). Similar structures are found at the margins of the developing hemi-neuromeres, occasionally also basally within the latter (Figure 11). These chromatin-rich bodies are neither labelled by the PH3 antibody, nor do they exhibit the typical tubulin-positive labelling of the surrounding cytoskeleton. For this reason, we assume that they represent pycnotic bodies related to cell death.

From ES 5 onwards, the hemi-ganglion anlagen of each segment are virtually touching at the midline (Figures 2I, 5C,D, 8 and 9). Some of the VMR cells have remained in an apical position (see below). The other part is wedged between the basally growing hemi-ganglion anlagen, being medio-laterally compressed and often unpaired (Figures 2I, 8A’,B’ and 9B–C’). Judging from the position and flattened appearance, these latter cells have differentiated into glia. Furthermore, some of the basal-most cells show distinct cytoskeletal tubulin labelling and are in close contact with the first transversally extending axonal growth cones of the segmental commissures during ES 7 (Figure 8A”).

#### Epidermal cells originate from the VMR and the ventro-lateral ectoderm

The few remaining apical cells of the VMR differentiate into epidermis cells. From ES 5 onwards, they start to become increasingly distinct from the enlarging NSCs and interspersed flask-shaped cells within the central invaginations. They are arranged in an irregular longitudinal ‘row’ that is one to two cells wide (Figures 2F, 5C,D, 8A,B,C and 9). The ectodermal cells lying directly anterior, lateral and posterior to each invagination adopt epidermal fate as well. As soon as the central invagination has formed in each hemi-segment, these cell regions start to become morphologically distinguishable based on a comparatively intense staining of their smaller oval nuclei (Figure 2F,I). With ongoing enlargement of the NSCs, the small epidermis cells can be easily identified (Figures 5D, 8A,B,C, 9 and 10B). They come to lie on top of the peripheral-most NSCs of the hemi-ganglion anlagen. Thus, the entire rim of the deepened invagination is apically covered by the prospective epidermis in late embryonic stages. The apical closure of the epidermis takes place during post-embryonic development only (data not shown).

### Cell counts in hemi-neuromeres of walking leg segment 1

**Figure 12:**
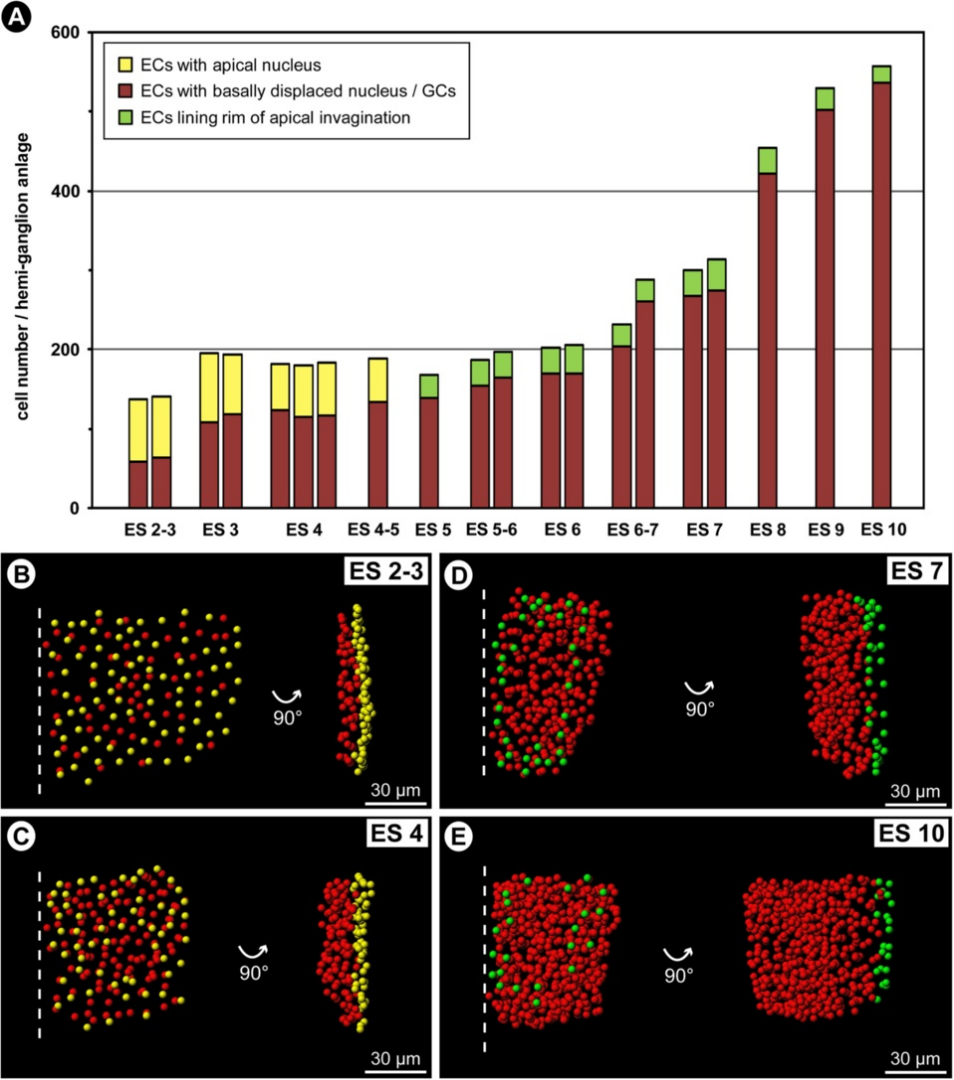
**Cell counts in walking leg neuromere 1 of *Pseudopallene *sp. (late ES 2 – ES 10).** Each bar stands for an analysed specimen. Immigrating cells with basally displaced nucleus as well as already detached neural cells (including all types of NPs) are shown in dark red. Cells with apical nucleus during early neurogenesis are labelled in yellow. Cells at the rim of the central invagination with unclear epidermal or neural fate are shown in green. **(A)** Overview of counted cell numbers per hemi-neuromere over the course of development. Note the slow increase of immigrating/detached GCs up to ES 6 and their still considerable increase during the second phase of embryonic neurogenesis. **(B–E)** Spots-model of selected hemi-neuromere counts of different developmental stages. Ventral view shown to the left, medial view to the right. Note hemi-neuromere compaction along the medio-lateral axis during development **(B–D)**. During the second phase of embryonic neurogenesis, the apico-basal extension of formed hemi-ganglion anlage increases dramatically **(D,E)**.

Complete cell counts in the hemi-neuromeres of walking leg segment 1 (including the VMR) were performed in all relevant stages of embryonic development (Figure 12). Obtained cell numbers were used to elucidate i) for how long new cellular material is added to the nervous system and ii) whether a significant increase of cell numbers can be detected in any of the developmental phases.

In late ES 2, the hemi-neuromere of walking leg segment 1 comprises slightly less than 140 cells. From ES 3 onwards, the overall cell number has increased to approximately 200 and stays in a remarkably similar range up to ES 6, where it barely exceeds 200. This is surprising, in light of the ongoing cell divisions in the apical VNE throughout these developmental stages. The high number of cells in ES 3 compared to the following stages could be the result of ambiguity of cell identity at the margins of the hemi-neuromere so that several non-VNE cells might have been included in the count of this early stage. However, also from ES 4 onwards, no clear trend towards an overall increase of cell numbers is detectable. A likely explanation for this seems to be the documented cell death at the peripheral margins of the hemi-neuromere, especially close to and within the VMR. From late ES 2 to ES 6, comparison of the ratio and absolute numbers of VNE cells with apical nucleus on the one hand (Figure 12A–C, yellow) and immigrating cells on the other (Figure 12A–C, red) shows a gradual but continuous increase of the latter and a decrease of the former. In line with the observations on the morphological processes of early neurogenesis, this supports a gradual and sequential immigration of cells rather than any partially synchronized cell immigration in distinct temporal ‘waves’ or ‘pulses’.

From ES 6 onwards, a distinct increase of cell numbers is observable in the hemi-ganglion anlagen of walking leg segment 1 (Figure 12A,D,E). In ES 8, the overall cell number has already more than doubled (~450 cells) and eventually approaches 550 cells in a pre-hatching ES 10. The small amount of apical rim cells (Figure 12A,D,E; green) that could not be assigned with absolute certainty to epidermal or neural fate decreases during later stages, because the apical hemi-neuromere portion comprising the NSCs becomes more distinctly set off from the surrounding smaller epidermis cells. Notably, the distinct increase of cell numbers from late ES 6 onwards shows a temporal correlation to the differentiation of the NSCs. Coupled to the observed mitotic activity of the NSCs and sub-apical INPs during this developmental phase, the cell increase in the underlying ganglion anlagen lends further support to a significant contribution of both cell types to GC generation during advanced embryonic stages.

### Preliminary data from other pycnogonid species

#### Early neurogenesis with groups of immigrating cells

**Figure 13:**
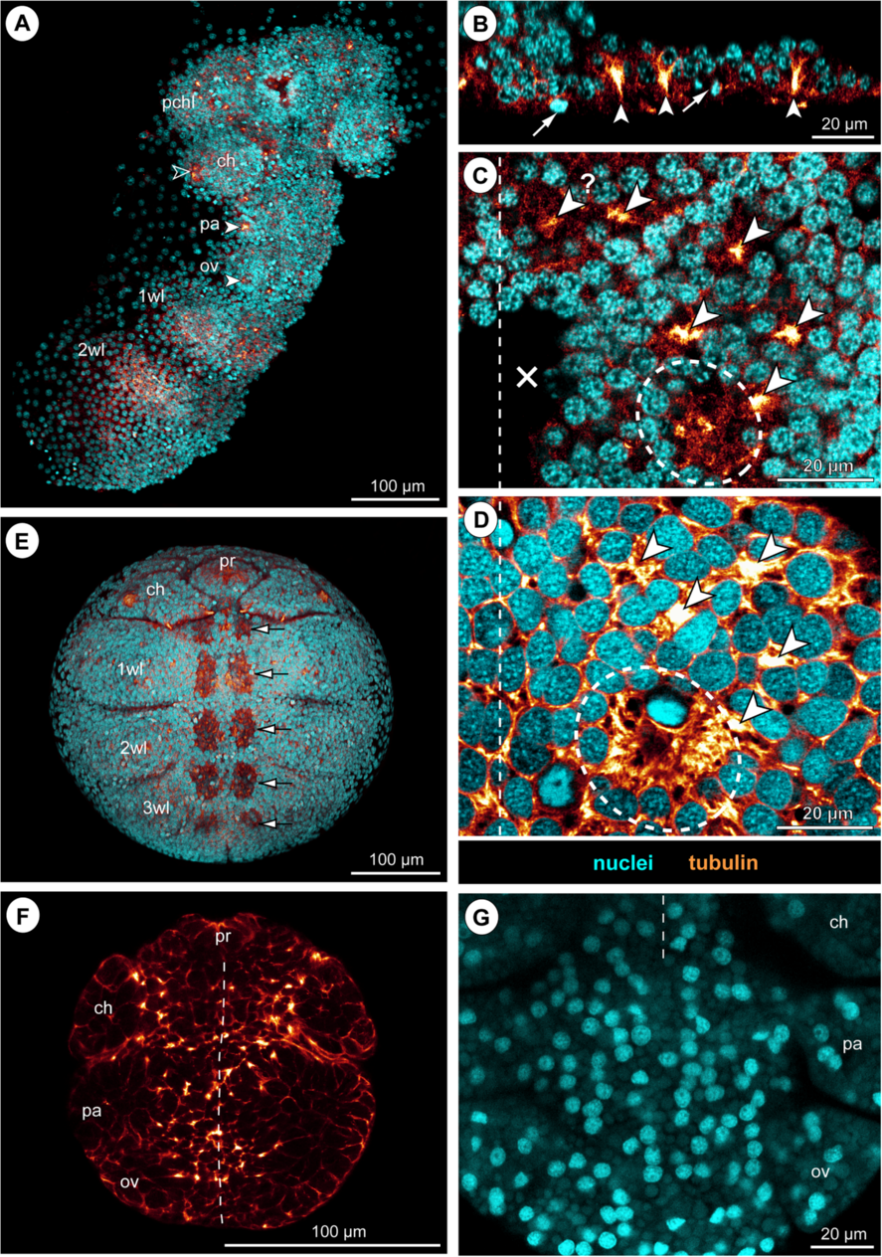
**Early neurogenesis in *Callipallene *sp. and *Pycnogonum litorale*.** Tubulin labelling and/or nuclear counterstain of embryos of *Callipallene* sp. **(A–C,E)**, *Pseudopallene* sp. **(D)** and *Pycnogonum litorale***(F,G)**. Dashed vertical lines mark the VMR. **(A)** Ventral overview of germ band, Imaris volume. Morphologically left half of the post-cheliforal region is missing (dissection damage). Note bright orange dots of CISs in pre-cheliforal lobes and in parts of the VNE. Open arrowhead indicates immigrating cells of the forming spinning gland. Solid arrowheads mark conspicuous sites of cell immigration lateral to the palpal and ovigeral hemi-neuromeres. **(B)** Transverse optical section through pre-cheliforal lobe. Arrowheads mark CISs. Arrows indicate apical mitoses. **(C,D)** Comparison of CIS pattern in walking leg 1 hemi-neuromeres of *Callipallene* sp. (**C**, same stage as in **A**) and *Pseudopallene* sp. (**D**, ES 4). Note similar number and arrangement of CISs (arrowheads) and the beginning formation of an invagination in the posterior region (stippled ovals). Arrowhead with question mark in **C** marks CIS with no potential counterpart in **D**. The VNE cells of *Callipallene* sp. are smaller and more numerous. Cross in **C** marks damaged region. **(E)** Ventral overview of embryo in advanced stage of neurogenesis, Imaris volume (blend). Arrows mark segmental invaginations of the ovigeral and the four walking leg neuromeres. **(F,G)** Overviews of the VNE of *P. litorale*, stage 4 according to Machner and Scholtz [122], horizontal 2D projection of curved composite optical sections. Note numerous tubulin-labelled apical spots in **(F)**. The apical VNE of the embryo has a remarkably low number of cells (as indicated by the few nuclei in **G**). Less intensely stained lipid drops (embryonic yolk) are interspersed between the nuclei.

*Callipallene* sp. is another callipallenid representative with embryonized development. Embryos in an early stage of embryonic morphogenesis show distinct tubulin-labelled spots in the neuroectoderm, most clearly in the pre-cheliforal lobe and cheliforal neuromere but also further posteriorly in the VNE (Figure 13A–C). As in *Pseudopallene* sp., these spots relate to bundled apical cell processes of CISs that comprise only few cells (~5, Figure 13B). In the hemi-neuromeres of walking leg segment 1, five to six CISs are positioned in the anterior region, whereas first signs of a nuclei-free invagination are detectable posteriorly (Figure 13C). The anterior CISs of *Callipallene* sp. show a similar spatial arrangement as in *Pseudopallene* sp., but are not as closely spaced, being separated by two to three apical cells (Figure 13C,D). During the later stages of embryonic morphogenesis, paired central invaginations form in all four walking leg neuromeres (Figure 13E).

In *Pycnogonum litorale*, embryonic development terminates with the hatching of a protonymphon larva that bears only three pairs of limbs, the chelifores and the palpal and ovigeral larval limbs [122,123]. Tubulin labelling also reveals in this species distinct spots relating to apically attached cell processes wedged between chelifore and proboscis anlagen and in the VNE of the palpal and ovigeral segment anlagen (Figure 13F). In contrast to the callipallenid representatives, the embryonic VNE of *P. litorale* comprises remarkably few cells (Figure 13G), which contain small yolk/lipid droplets (unspecifically labelled in Figure 13G; see also [124] for *Achelia echinata*). Therefore, assessment of cell shapes in the more intricate pattern of cytoskeletal tubulin labelling proves difficult and F-actin staining by phallotoxins consistently fails in the embryonic stages. For these reasons, it remains currently unresolved whether single cells or small groups of cells immigrate at the spots marked by high tubulin signals.

#### Advanced neurogenesis with NSCs

**Figure 14:**
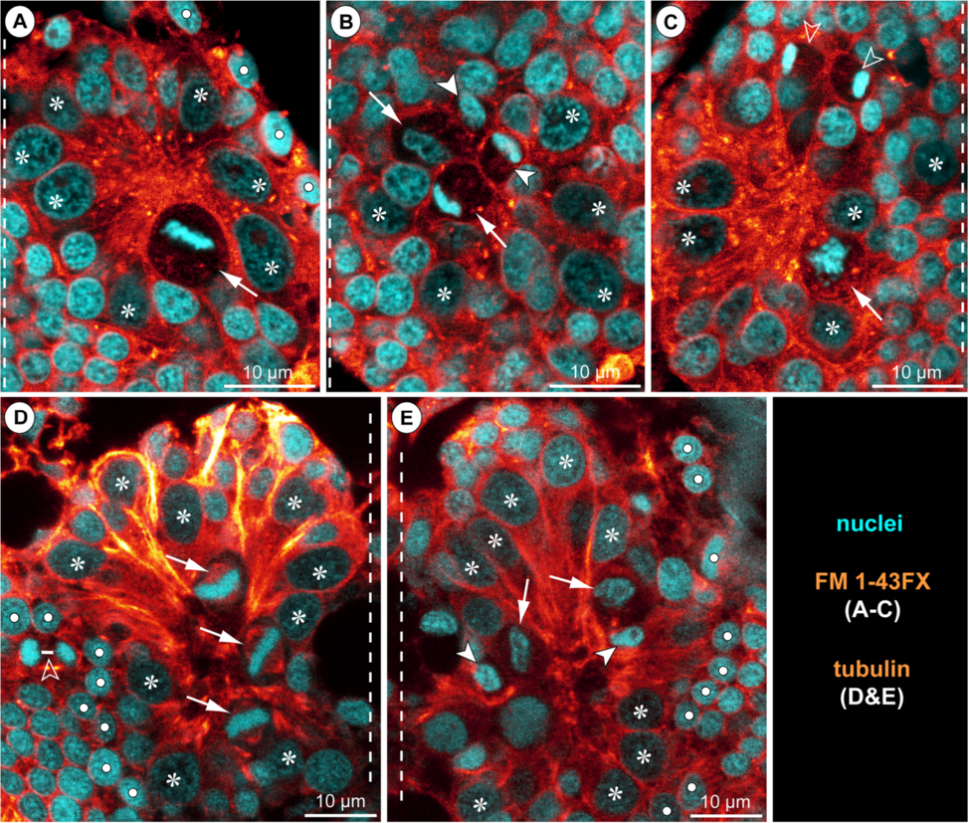
**NSCs during advanced neurogenesis of *Stylopallene cheilorhynchus *and *Nymphon gracile*.** Optical sections of FM 1-43FX-labeled specimens of *Stylopallene cheilorhynchus***(A–C)** and tubulin-labelled specimens of *Nymphon gracile***(D,E)** with nuclear counterstain. Oblique slicers have been aligned to show asymmetry/symmetry of forming sister cells as clearly as possible. Therefore, sections are frequently oriented obliquely horizontal. Dashed vertical lines mark the VMR. Asterisks label large NSCs that are not in division. White spots indicate epidermal cells. **(A–C)** Walking leg ganglion 1 of PS 1. **(A)** Apical section through morphologically left hemi-ganglion anlage. Note significant size differences between NSCs and all other cells. Arrow points at NSC in metaphase, note off-centre position of the metaphase plate. **(B)** Slightly basal to **A**. Two asymmetrical NSCs divisions are marked. Arrows mark the larger, arrowheads the smaller of the newly forming sister cells. **(C)** Section through morphologically right hemi-ganglion anlage. Arrow points at NSC in prophase. Open arrowheads point at forming sister cells of a symmetrical division of an INP amongst the more peripheral GCs. **(D,E)** Walking leg ganglion 1 of PS 2. **(D)** Apical section through morphologically right hemi-ganglion anlage. Arrows indicate three NSCs in metaphase (~tangential orientation). Open arrowhead marks smaller symmetrical division (telophase) of an epidermal precursor bordering the hemi-ganglion anlage. **(E)** Apical section through morphologically left hemi-ganglion anlage. Two asymmetrical NSC divisions are marked. Arrows mark the larger, arrowheads the smaller of the newly forming sister cells. Bluish halo on the right side represents autofluorescence of the larval cuticle.

The walking leg-bearing larva of the callipallenid *Stylopallene cheilorhynchus*[98] shows a similar degree of trunk and VNC differentiation as the hatching stage of *Pseudopallene* sp. The hatching larva of *S. cheilorhynchus* features conspicuous large cells at the apical side of the developing ventral ganglia, which correspond to the NSCs in advanced neurogenesis of *Pseudopallene* (Figure 14A–C). NSC nuclei in interphase are less intensely labelled (Figure 14A–C) and their divisions are asymmetrical (Figure 14B), first signs of this asymmetry being already discernible in the off-centre position of the metaphase plate (Figure 14A). Basal to the apical region comprising the NSCs, some symmetrical divisions of smaller cells were detected (Figure 14C).

Similar findings were made in *Nymphon gracile*. This species hatches as a protonymphon larva without any external anlagen of walking legs and without any significantly enlarged neuroectodermal cells (data not shown). In post-embryonic stage 2, minute limb buds of walking leg pair 1 have formed and at the apical side of the affiliated ganglion anlage large and mitotically active NSCs have differentiated (Figure 14D,E). Asymmetrical NSC divisions are detectable (Figure 14E), with tangential to slightly oblique orientation.

## Discussion

### The two-phase character of early and advanced pycnogonid neurogenesis

**Figure 15:**
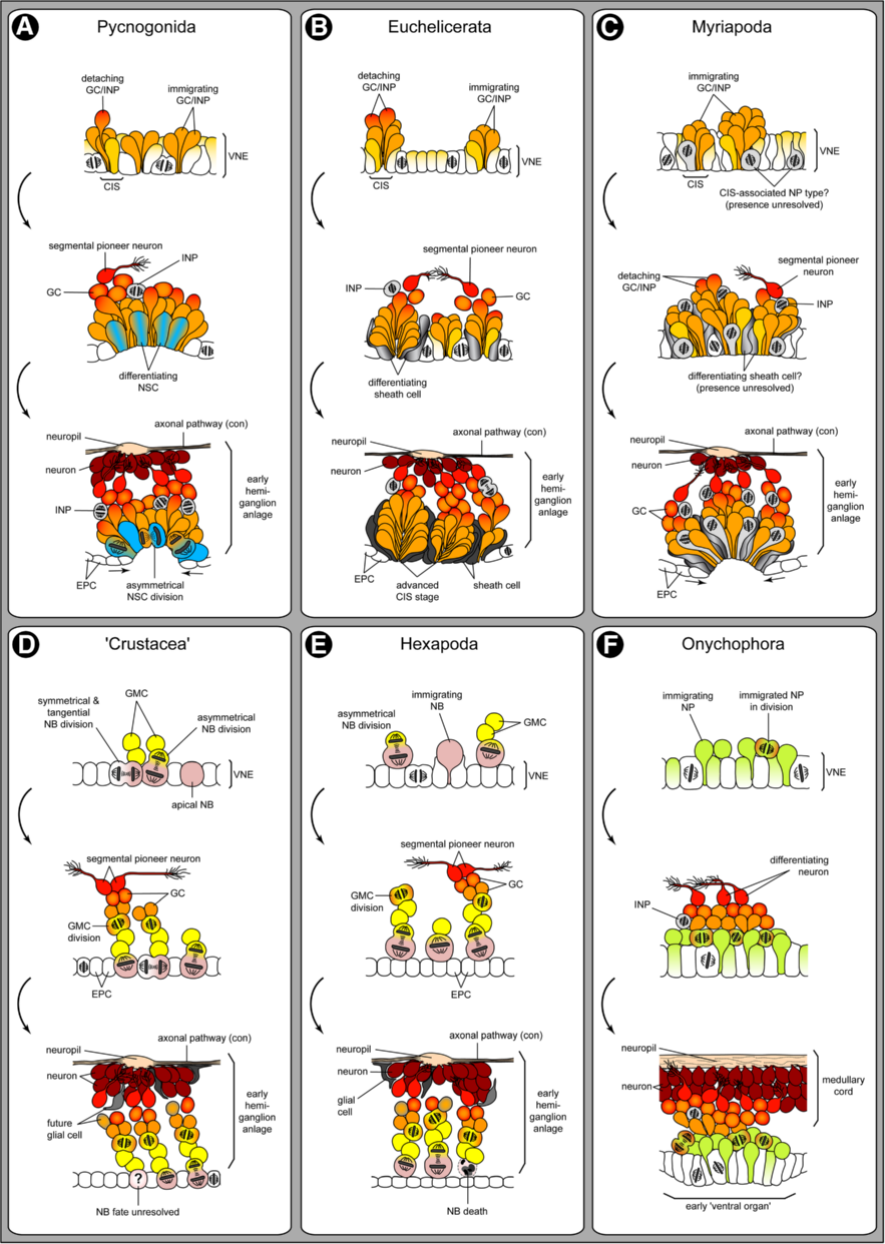
**Comparison of neurogenesis in major arthropod lineages and Onychophora.** Schematic sagittal sections through single hemi-neuromeres. For simplicity, only three CISs/NBs are depicted. Different colours (blue, yellow, pink, green) have been used to highlight well-characterized NP types. Insufficiently characterized NP types are depicted in light grey (indicating not necessarily homology across taxa). **(A)** Pycnogonida (predominantly based on *Pseudopallene* sp.). Few CISs form within the VNE. Unordered, tangential cell divisions occur in the VNE. Single GCs/INPs detach basally, forming a loose layer, in which first neurons start to differentiate. Apically, a central invagination forms, deepening with ongoing development. Large spindle-shaped NSCs differentiate and divide in tangential or slightly oblique orientation. Their smaller daughter cells immigrate independently. Sub-apical INPs divide in the anterior and posterior portions of the hemi-ganglion anlage. Prospective epidermis cells cover the invagination’s rim and later overgrow it. **(B)** Euchelicerata (predominantly based on *Cupiennius salei*). CISs form sequentially in the VNE. Unordered, mostly tangential cell divisions occur in the VNE. Mostly immature GCs detach and differentiate basally to the CISs. Several CISs transform into cell-rich units, being enclosed by glial-like sheath cells (representativeness for euchelicerates unclear). Scattered symmetrically dividing INPs are found close to the forming neuropil. The epidermis overgrows the hemi-neuromeres apically. **(C)** Myriapoda (predominantly based on *Glomeris marginata*). Similar to euchelicerates, but VNE cells are more closely packed. Spatial correlation of cell divisions with CISs is observable, but existence of a specialized NP remains unresolved. In advanced stages, a central invagination forms, being later overgrown by the epidermis. Spindle-shaped NPs and sub-apical NPs occur, but cell types and division patterns await reinvestigation. **(D)** ‘Crustacea’ (predominantly based on *Orchestia cavimana*). NBs form sequentially in a stereotyped division sequence. They are maintained apically and produce GMCs by asymmetrical radial divisions. Some NBs intermittently switch to symmetrical tangential divisions to generate epidermal cells. GMCs divide (typically) once to produce immature neurons and/or glial cells. NB fate remains unresolved. **(E)** Hexapoda (predominantly based on *Drosophila melanogaster* and *Schistocerca* sp.). Similar to crustaceans, but sequentially specified NBs immigrate into sub-apical position prior to proliferation. Most NBs undergo apoptosis after generation of their lineage. Association of NBs with sheath and cap cells has been reported in grasshoppers only, their plesiomorphic character being therefore questionable and here omitted. **(F)** Onychophora (predominantly based on *Euperipatoides kanangrensis* and *E. rowelli*). NPs immigrate individually from the VNE without recognizable spatio-temporal pattern. Immigrated NPs form a sub-apical layer and divide at least once in symmetrical fashion, producing immature neurons (potentially also glial cells) and presumably some INPs. First neurons differentiate basal-most and extend growth cones anteriorly. In advanced stages, segmental ‘ventral organs’ develop as transformations of the VNE.

Embryonic neurogenesis of *Pseudopallene* sp. is here sub-divided into two phases (Figure 15A).

In an initial phase, predominantly post-mitotic cells immigrate from the VNE. During a certain time span, cell immigration occurs in defined CISs. With the applied techniques, we could recognize a stereotyped spatial pattern for at least some of the identified CISs. Additional cell material is generated by unordered tangential divisions in the apical VNE layer. In a second phase, a central invagination forms in each hemi-segment, being lined by enlarging NSCs. These are characterized by high mitotic activity and an asymmetrical mode of division. NSC divisions are mostly tangential or with a slightly oblique inclination. In between the NSCs, the generated smaller cells appear to immigrate basally, at least some of them representing INPs that divide morphologically symmetrically. Importantly, these two phases of neurogenesis are not strictly separated, the differentiation of the apical NSCs overlapping with the still ongoing immigration of early flask-shaped cells.

Our available data on other pycnogonid representatives – albeit still fragmentary – indicate similar neurogenic phases as shown for *Pseudopallene* sp. The early neuroectoderm of *Callipallene* sp. is characterized by the presence of CISs and the distinct tubulin spot pattern in the VNE of the indirectly developing *P. litorale* speaks as well for an early process involving cell immigration. In further support of such a first neurogenic phase as a pycnogonid-wide phenomenon, older studies describe a general ‘thickening’ of the ectoderm as the first sign of the developing neuromeres during pycnogonid embryology [93-95,124], although the exact processes leading to this initial thickening were not resolved. Only in a single study on *Callipallene emaciata*[96] were large NSCs (‘neuroblasts’) described for the very beginning of neurogenesis and claimed to produce GCs by repeated divisions. Clearly, this description cannot be substantiated by our new data. It seems probable that in the older study [96] observations on later stages of neurogenesis have been erroneously extrapolated into the initial neurogenic phase of *Callipallene emaciata*. The earliest depicted stage shows already smaller GCs basal to large NSCs (Figure four point one, p. 33 in [96]) and the initial neurogenic processes have evidently been missed. Considering the difficulties we encountered to identify CISs during analysis of histological sections alone, it is not surprising that they were not noted in the previous studies.

A later second neurogenic phase with NSCs as in *Pseudopallene* sp. is not only apparent in *Stylopallene cheilorhynchos* (as an additional callipallenid representative) but also in *Nymphon gracile*. Based on the latter finding, a causal relation between the differentiation of NSCs and the embryonized development of callipallenids can be excluded. Instead, apical NSCs (and sub-apical INPs) are indicated to be a characteristic feature of a second phase of neurogenesis in pycnogonids as a whole, regardless of the developmental modes in the different groups. This conclusion is in good agreement with older studies. All data covering pycnogonid neurogenesis in the corresponding advanced developmental stages report large proliferating NSCs that line paired segmental invaginations at the apical side of the ganglion anlagen, the so-called ‘ventral organs’ [93-96,125,126]. Almost unanimously, the NSCs have been interpreted as teloblast-like neural precursors or ‘neuroblasts’. Only Sanchez [95,126] assigned them a neurosecretory instead of a neurogenic function. While Morgan [93] could detect no predominant direction of the NSC divisions, Winter [96] reports mostly radial spindle orientations. According to him, this leads to the formation of radial rows of GCs at the basal side of the NSCs. In contrast to this, we found that the NSC divisions are mostly oriented tangentially or with a slightly oblique inclination. Notably, however, with ongoing deepening of the central invaginations, assessment of the orientation of divisions becomes problematic within the increasingly three-dimensional cell arrangements and the accompanying morphological changes in the VNE. Due to the invagination process, the apical and basal poles of the cells in the peripheral portions of the invagination cease to be aligned with the apico-basal axis of the ventral embryonic hemisphere. Dividing cells close to the rim of an invagination can thus be seen as exhibiting radial-like spindle orientation in relation to the embryonic axes. However, when taking the inwards-outwards directed apical and basal cell poles in the invagination as reference, this might more correctly be designated as tangential division. The latter interpretation is here preferred, since the polarity of a cell in its local environment seems to be the appropriate reference system.

In our study on *Pseudopallene* sp., we document for the first time the morphologically asymmetrical divisions of the pycnogonid NSCs and additionally identify a so far unrecognized INP type that divides basal to the NSCs. The morphological asymmetry of the NSC divisions represents one of the important arguments in support of their stem cell-like nature, which means that the cells self-renew during each division at least during a certain developmental time span. Further support for this view is provided by the relative constancy in their numbers during late embryonic and early post-embryonic development (ES 7 – PS 1), despite their high proliferation activity and the constantly increasing number of the underlying GCs.

### Pycnogonid neurogenesis compared to other arthropod taxa and Onychophora

Most recent contributions comparing neurogenesis among the arthropod lineages have pointed out the two distinct modes involving either spatially stereotyped CISs (in euchelicerates and myriapods) or specialized NSCs (NBs in hexapods as well as in malacostracan and branchiopod crustaceans) [18-20,65,81,121,127,128]. In some recent discussions, however, the occurrence of different modes of neurogenesis in spatially separate regions in a single organism have been highlighted [20,36]. This includes, for instance, the formation of the pars intercerebralis and pars lateralis in the brain of *Drosophila melanogaster* via groups of immigrating cells rather than NBs [129] as well as the direct immigration of immature neurons pioneering the protocerebral commissure in the brain of *Schistocerca gregaria*[130]. Similarly, development of the (peripheral) stomatogastric nervous system of insects is characterized by immigration of groups of cells from a placode in the dorsal stomodeum wall [131]. These phenomena have been used to argue for CISs as the plesiomorphic condition within arthropods. Compared to the other lineages, one of the most intriguing features of pycnogonid neurogenesis is the formation of CISs and of specialized NSCs in exactly the same neuroectodermal region, but in subsequent phases. This illustrates that neurogenesis in a given region does not necessarily have to follow the same mode throughout development of an organism.

#### A terminological suggestion: cell internalization site

In general, the initial immigration of flask-shaped cells in specific sites of the VNE of each hemi-segment as seen in *Pseudopallene* sp. (and other pycnogonids) shows unmistakable similarities to the processes of early neurogenesis in euchelicerates (Figure 15B, spiders: [14,58,64,76,132,133], xiphosurans: [59,60]) and myriapods (Figure 15C, centipedes: [57,62,63], millipedes: [61,64], symphylans: [76]). These specific sites of cell immigration have been termed ‘invagination sites’ [58] or ‘immigration sites’ [60]. Yet, the term ‘invagination’ is confusing with respect to later processes of neurogenesis, during which the complete central area of a hemi-neuromere may sink in to from a conspicuous invagination [60,120]. Some authors also claim that ‘immigration’ fails to characterize the early processes correctly, suggesting instead ‘ingression’ [65,76]. However, since both terms describe an interiorly directed movement of a cell without direct involvement of a division, they are here considered as representing synonyms. For the sake of terminological consistency, ‘immigration’ has been applied in this study with respect to the interiorly directed movement of cells. Nonetheless, it has to be noted that in some euchelicerate and myriapod representatives, tiny local pits (‘invaginations’) are indeed observed in the sites where several adjacent apical cells are in the process of immigration (see [108,109,134] for spiders). To acknowledge this mixture of phenomena and avoid terminological conflicts, the neutral term ‘cell internalization site’ (CIS) is here given preference and suggested for future studies.

#### The slow ‘crystallization’ process of CISs in *Pseudopallene* sp

In *Pseudopallene* sp*.*, the earliest phase of neurogenesis from the slow stratification of the VNE via basal displacement of nuclei up to the emergence of recognizable CISs comprising groups of flask-shaped cells extends over several embryonic stages. Contrary to this, no such slow and gradual development of CISs has been reported in any euchelicerate or myriapod representative. Two possible explanations for this circumstance are feasible.

##### Embryonic development of pycnogonids is slow compared to development of the studied euchelicerates and myriapods

The complete embryonic development of *Pseudopallene* sp. has been estimated to take between 1 and 2 months at 15°C in culture, a water temperature corresponding to the natural habitat during the reproductive season (13–15°C, unpublished data). By comparison, in *Pycnogonum litorale*, a representative that hatches as small protonymphon larva without any post-ovigeral trunk segments [122,123], embryonic development alone lasts about three weeks at 15°C and the post-embryonic anamorphic development of this species takes additionally several months [123,135]. As a consequence of the slow development of pycnogonids, a finer resolution of the successive basal displacement of cells belonging to prospective CISs may be achievable than in fixed material of the faster developing spiders or myriapods investigated with similar techniques (~14 days embryonic development of *Lithobius forficatus* at 25°C [62], ~48 days of embryonic development of *Strigamia maritima* at 13°C (almost half of the time in early cleavages, germ band development far more rapid) [136], ~7–8 days embryonic development of *Parasteatoda tepidariorum* at 25°C [109], approximately twice as long in *Cupiennius salei*).

##### No focus on the formation process of single CISs in previous investigations

Studies on euchelicerates and myriapods have mainly focused on the number and arrangement of CISs and their composition and structure as soon as they have become recognizable in the VNE [58,60-62]. However, the exact formation process of single CISs *per se* was never documented in detail. Accordingly, it is feasible that a faster but nonetheless gradual basal displacement of cells (as opposed to a concerted simultaneous immigration) in the regions of the prospective CISs does occur in euchelicerates and myriapods but has not been resolved.

#### CIS number and arrangement in *Pseudopallene* sp. compared to euchelicerates and myriapods

**Figure 16:**
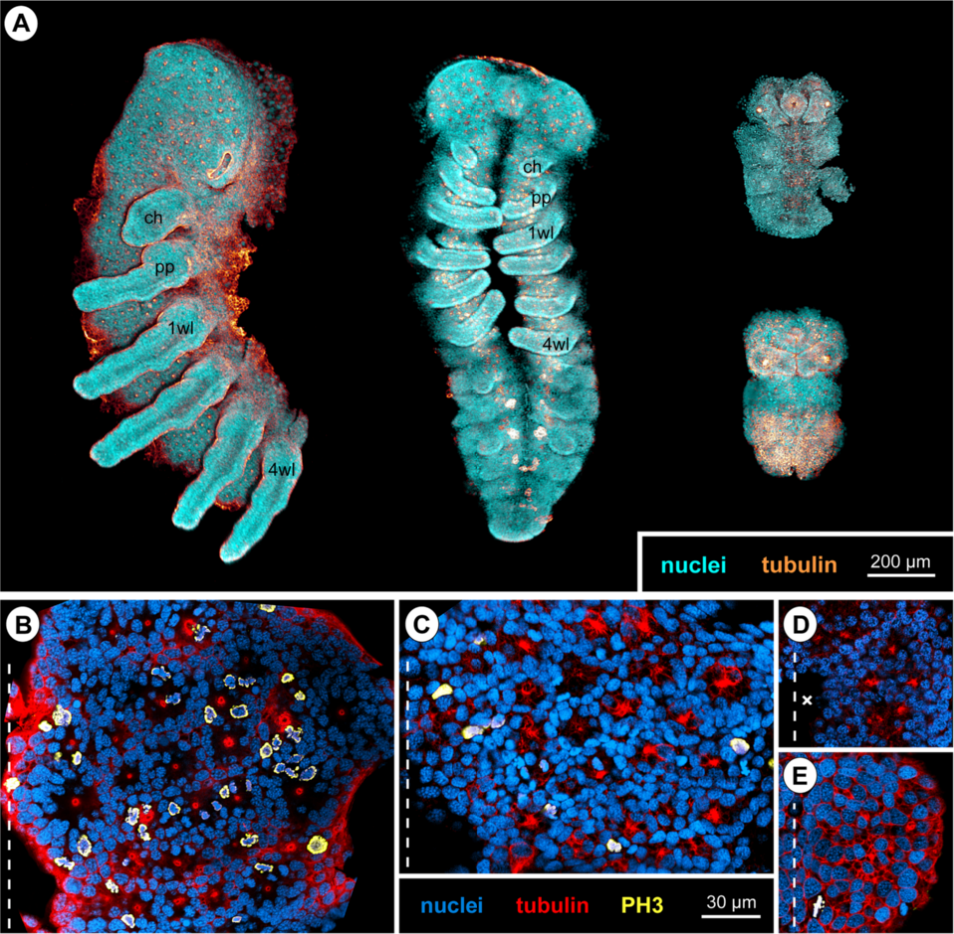
**Comparison of germ band size, cell number and cell sizes between callipallenid pycnogonids and spiders.** Tubulin-labelled embryos with nuclear counterstain, in part with additional PH3 labelling. **(A)** Ventral view of germ bands of *Cupiennius salei* (left, only left half of prosomal region shown) and *Parasteatoda tepidariorum* (middle) compared to *Callipallene* sp. (right-top) and *Pseudopallene* sp. (right-bottom), flat preparations, Imaris volumes. **(B–E)** Apical horizontal sections (2D projections of curved composite optical sections). Dashed line marks the VMR. **(B)***C. salei*, hemi-neuromere of walking leg segment 2. Note the apical mitoses scattered between the CISs. **(C)***P. tepidariorum*, hemi-neuromere of walking leg segment 2. Note some scattered apical mitoses and the distinct tubulin labelling of CISs. **(D)***Callipallene* sp., hemi-neuromere of walking leg segment 1. The apical area of the hemi-neuromere represents not even a quarter of the area in the corresponding hemi-neuromeres of the two spider species. Neuroectodermal cell size appears similar to spiders. Cross marks damaged region. **(E)***Pseudopallene* sp., hemi-neuromere of walking leg segment 1. The apical area of the hemi-neuromere is significantly smaller and the neuroectodermal cells are slightly larger than in the two spider species.

In all recently studied euchelicerates and myriapods, 30 or more CISs are formed per ventral hemi-neuromere, being at least transiently arranged in a grid-like pattern that in many cases comprises seven roughly transverse rows (Figure 16B,C, Euchelicerata: *Cupiennius salei*[58,64], *Parasteatoda tepidariorum*[64], *Limulus polyphemus*[60]; Myriapoda: *Ethmostigmus rubripes*[57], *Lithobius forficatus*[62], *Strigamia maritima*[63], *Glomeris marginata*[61,64], *Archispirostreptus* sp. [137]). Since the NBs in hexapods and some crustaceans are also arranged in a similar grid-like array (Hexapoda: *Locusta migratoria*[28], *Schistocerca americana*[31], *Drosophila melanogaster*[138,139], *Ctenolepisma longicaudata*[34]; Crustacea: *Diastylis rathkei*[32], *Cherax destructor*[33], *Daphnia magna*[37]), the general pattern has been suggested to be a conserved character of arthropod neurogenesis [64,121,127,128], and might be controlled among others by early embryonic axis patterning genes [140-142].

In contrast to euchelicerates and myriapods, a maximum of only eleven very closely spaced and stereotypically arranged CISs could be identified per hemi-neuromere of walking leg segment 1 in *Pseudopallene* sp. A comparably low number of CISs was found in a corresponding hemi-neuromere of *Callipallene* sp. Owing to this low CIS number, no distinct grid-like array with seven roughly transverse rows is recognizable.

Furthermore, formation of the complete set of ≥30 CISs in euchelicerates and myriapods occurs sequentially within each hemi-neuromere [58-62], having been sometimes described to involve distinct formation ‘pulses’ of subsets of CISs [58,59,61]. In *Pseudopallene* sp., neither temporally distinct ‘pulses’ of CIS formation nor sequential emergence of CISs could be observed.

In comparison to the well-investigated euchelicerate (Figure 16) and myriapod representatives, the pycnogonid VNE encompasses only a small area coupled to a low overall cell number. This alone could account for the apparent lack of additional CISs in extant pycnogonids. In between the already closely spaced CISs, additional ones would need to recruit even more – but non-existent – cellular material from the apical neuroepithelium. On an evolutionary scale, it can therefore be speculated that the formation of NSCs in the second neurogenic phase of pycnogonids represents a derived developmental mechanism that enables generation of additional GC material in a small VNE area with too few cells for additional CISs. Interestingly, a correlation between the mode of neurogenesis and VNE morphology has been discussed recently in the context of the evolution of NBs in the tetraconate lineage [127].

#### Pycnogonid NSCs versus tetraconate NBs – common origin or convergent evolution?

The asymmetrically dividing NSCs that enlarge during the second phase of pycnogonid neurogenesis are at least in some aspects reminiscent of crustacean and hexapod NBs (see [39] for general definition). Yet, how many detailed similarities beyond size and division mode provide arguments for a common origin of both neural precursor types?

##### Pycnogonid NSCs share with crustacean NBs the apical position in the VNE

**Figure 17:**
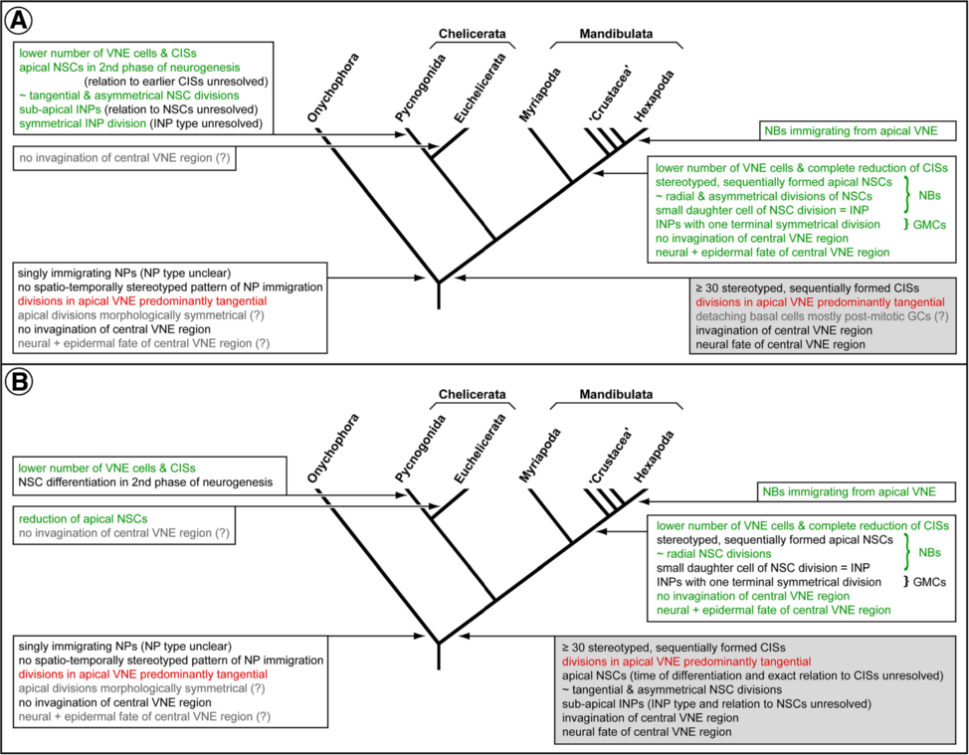
**Two scenarios on the evolution of arthropod neurogenesis.** Both scenarios are set against the backbone of the Chelicerata (Pycnogonida + Euchelicerata) and the Mandibulata (Myriapoda + Tetraconata) hypotheses. Onychophora are taken as the out-group but do not help significantly in the establishment of the arthropod stem species pattern. Colour code of characteristic features: green = apomorphic, red = plesiomorphic, black = apomorphic vs. plesiomorphic unclear, grey = in need of verification. Grey box at the bottom shows the reconstructed features in the arthropod stem lineage. **(A)** Convergent evolution of pycnogonid NSCs and tetraconate NBs. **(B)** Common origin of pycnogonid NSCs and tetraconate NBs from an ancestral NSC type with subsequent lineage-specific evolution of the latter. The critical assumption in this scenario is the occurrence of NSCs in myriapods.

Similar to the NSCs of pycnogonids, malacostracan and branchiopod NBs are maintained within the apical VNE during their proliferation activity (Figure 15A,D) [26,27,32,33,35-37,143-146]. Notably, this similarity only holds, if the inner surface of the invaginations in pycnogonids is considered as being apical; a view that we prefer (see Discussion above). This apical position contrasts to hexapod NBs, which individually immigrate and detach from the apical neuroepithelium before starting proliferation in a sub-apical layer (Figure 15E) [24,25,28,30,31,34,147-149]. Several publications have favoured the hexapod mode of NB immigration as representing the plesiomorphic condition of tetraconates [35-37,65]. If true, apically maintained crustacean NBs would be an apomorphic feature that cannot be used for phylogenetic comparisons with non-tetraconate arthropods. However, in virtually all recent phylogenetic analyses, crustacean paraphyly is strongly supported. Here, either branchiopods, or sometimes also malacostracans are indicated as being more closely related to hexapods (e.g. [52-54,56,87,150] or [23], respectively). A notable exception is found in a comprehensive molecular study [51] in which malacostracans and branchiopods are recovered together in a monophyletic sub-group within paraphyletic crustaceans. Against the phylogenetic backbone of this study, apically maintained NBs could still be interpreted as a derived feature of the malacostracan-branchiopod sub-group (at least as long as data on other crustacean groups are missing). Yet, in all other cases, the phylogenetic relationships imply either independent evolution of apically maintained NBs in the two crustacean lineages, or alternatively – and more parsimoniously – the immigration of hexapod NBs as being the derived tetraconate condition (Figure 17).

##### Pycnogonids and tetraconates share sub-apical INPs

The smaller sub-apical INPs of pycnogonids show a morphologically symmetrical mode of division (Figure 15A). Their correspondence to tetraconate GMCs (Figure 15D,E) [35,40] may be suggested. However, compelling evidence for this suggestion remains currently scarce. Neither the genealogical relationship of the sub-apical pycnogonid INPs to the apical NSCs, nor the number of divisions undergone by each INP could be established in our study. In contrast to these similarities there are a number of clear differences between pycnogonid NSCs and tetraconate NBs.

##### The two NP types do not share a corresponding time of differentiation

Tetraconate NBs emerge at the beginning of neurogenesis. Their proliferation activity generates the vast majority of future neural lineages (Figure 15D,E). The pycnogonid NSCs enlarge only after the initial phase of neurogenesis has already led to a considerable thickening of the VNE (Figure 15A).

##### The two NP types do not differentiate in a local environment with similar cell fate

Pycnogonid NSCs arise in an invagination in the centre of each hemi-neuromere, representing an ectodermal region that will adopt neural fate in its entirety. The mechanism underlying pycnogonid NSC specification within this neuroectodermal invagination remains, to date, unknown. Ventral epidermal cells have their origin in the more peripheral regions of the hemi-segment (Figure 15A). By contrast, tetraconate NBs differentiate in the apical ectodermal layer in between prospective epidermal cells (Figure 15D,E). Hence, tetraconate NBs differentiate in an ectodermal region with binary fate, although the NB specification processes differ between crustaceans and hexapods [33,37].

##### The two NP types do not share a similar spatial arrangement in the ventral hemi-neuromeres

In hexapods and apparently also in some crustacean taxa, the NBs are at least transiently arranged in a grid-like pattern in each hemi-neuromere [28,31,34,35,37,138,139]. A comparably distinct arrangement was not observed for pycnogonid NSCs, which differentiate only after the formation of the central invagination. Owing to the ongoing deepening and medio-lateral compression of the invagination, pycnogonid NSCs are found in a complex three-dimensional arrangement (but at least in some specimens, indications of bilaterally symmetrical arrangements were observed).

##### The two NP types do not share a corresponding orientation of their asymmetrical divisions

Pycnogonid NSCs are here reported to divide tangentially or with a slightly oblique inclination (Figure 15A). Due to this, the generated smaller daughter cells of pycnogonid NSCs are not automatically internalized during division but rather immigrate independently. By contrast, tetraconate NBs divide in radial direction (Figure 15D,E). Only in malacostracans and branchiopods, intermittent deviations from radial NB divisions are documented [32,37]. However, the resulting daughter cells appear to assume epidermal fate, whereas all cells generated in the central invagination of pycnogonids adopt neural fate.

In summary, apart from the comparably large size and asymmetrical division mode, only few additional similarities between pycnogonid NSCs and tetraconate NBs can be found. Hence, additional data is required for discussion and clarification of a possible homology of these cell types (see [151] for discussion of homology issues during developmental processes). In the future, more detailed studies are needed to better understand the characteristics and relationships of the pycnogonid NSCs and INPs. This includes a sound molecular characterization of the two NP types, *in vivo* proliferation experiments, and ideally even cell lineage studies. However, the establishment of a laboratory culture of a pycnogonid with embryonized development represents a pivotal prerequisite for such studies. Some of the important questions that need to be answered are:

i) What molecular mechanism underlies specification of NSCs in the pycnogonid VNE?

ii) Do the apical NSCs divide exclusively asymmetrically or do they switch intermittently to symmetrical divisions?

iii) Do all of the interspersed smaller apical cells and the sub-apical cells represent INPs or do they include already immature post-mitotic GCs?

iv) Does each sub-apical INP directly undergo terminal division or does it divide repeatedly?

Addressing these questions will help to evaluate potential similarities and differences and might lead to a conclusive hypothesis on the origin of tetraconate NBs and pycnogonid NSCs.

#### Myriapoda – a key in-group for the reconstruction of the evolution of arthropod neurogenesis

Following the discovery of a NSC type in pycnogonid neurogenesis, the cellular processes of neurogenesis in the putative sister group of the Tetraconata, the Myriapoda (Figures 1A and 17), should be re-evaluated. Recent studies on myriapods have revealed several notable characteristics of early neurogenesis. Initially, it shows distinct similarities to pycnogonids and euchelicerates, being characterized by the formation of a set of CISs in each hemi-segment (Figure 15C; centipedes: [57,62,63]; millipedes: [61,64]; symphylans: [76]). Yet, in contrast to the studied chelicerate representatives, there are indications of cell proliferation, which is spatially related to the CISs. By using BrdU (a marker of cells undergoing S-phase) in *in vivo* cell proliferation experiments, Whitington et al. [57] revealed single labelled cells within the CISs of the scolopendromorph *Ethmostigmus rubripes*. Furthermore, they reported dye-coupling of all cells of one CIS, which is also found in hexapod NBs and their early progeny (e.g., *Schistocerca gregaria*[130]). They concluded that each group of immigrating cells might represent a ‘proliferative unit’. Similarly, Dove and Stollewerk [61] describe in the VNE of the millipede *Glomeris marginata* apically dividing cells, which are slightly larger than the surrounding cells and are mainly positioned adjacent to or even within the CISs. These findings were interpreted in favour of a NSC type in the millipede VNE and of an ‘evolutionary intermediate’ mode of neurogenesis between chelicerates and tetraconates. Lastly, in the geophilomorph chilopod *Strigamia maritima*, each of the CISs comprises a conspicuous cell to which all other flask-shaped cells attach [63,128]. Again, this has been speculated to represent a first specialization of VNE cells during evolution ‘towards’ tetraconate NBs [20,65], but data on cell division patterns are so far unfortunately lacking in this species. Taken together, it is remarkable that indications of more ordered cell proliferation and potentially even specialized NPs (Figure 15C) have been presented in several recent investigations of early neurogenesis in different myriapod representatives.

Even more remarkable, however, are observations of advanced stages of myriapod neurogenesis, since they are clearly reminiscent of the processes in pycnogonids described here. The myriapod VNE of later stages is characterized by the formation of segmentally paired invaginations (Figure 15C) [57,63,137,152-156]. At least initially, an organization in separate CISs is still retained in these invaginations, the groups of flask-shaped cells radiating from its apical surface [57,63,137]. Yet, a detailed characterization of the occurring cell types in these developmental stages is unfortunately not available. In classical histological studies, however, the paired invaginations have been shown to be lined by an apical-most layer of spindle-shaped cells, which is basally contiguous with the underlying ganglion anlagen (scolopendromorph centipedes: [152]; scutigeromorph centipedes: [156]; symphylans: [153]; pauropodans: [154]). These apical cell regions have been named ‘ganglionic pits’ or ‘ventral organs’, in correspondence to the structures found in pycnogonids. Knoll [156] even claimed the spindle-shaped cells to be ‘neuroblasts’, due to their high proliferation activity (Figures forty-eight and forty-nine in [156]). Although not going as far in the interpretation, all other studies focusing on cell proliferation during this developmental phase report cell divisions amongst the spindle-shaped cells and/or the cells sub-apical to them [57,152-155]. Hence, the myriapod ‘ventral organs’ are clearly indicated as sources for additional GC material. Intriguingly, the location of cell divisions – especially as shown for pauropodans by Tiegs [154] – corresponds exactly to the ones of spindle-shaped NSCs and sub-apical INPs described here for *Pseudopallene* sp. In light of these correspondences, it is highly desirable that future work refocuses on myriapods. With the currently available data, it remains still impossible to prove or reject the existence of NSCs during any phase (early or advanced) of myriapod neurogenesis. Hence, the pending question of an ancestral NSC type of arthropod neurogenesis cannot – at least hitherto – be conclusively answered from arthropod in-group comparison and reconstruction alone.

#### Onychophora – key out-group for character polarization within arthropods?

Onychophora, or velvet worms, are nowadays strongly supported as close relatives to arthropods (Figures 1 and 17) [55,78,79,87] with which they form the Panarthropoda. A number of investigations have recently focused on the nervous system development in this arthropod out-group [20,76,157-159], aiming to uncover features of neurogenesis that allow clear character polarization within arthropods. However, although these studies agree in almost all aspects at the observational level (Figure 15F) [65], deviating interpretations of the involved cell types have been put forward. It is accepted that the first phase of onychophoran neurogenesis features the immigration of single NPs in a sub-apical position. In contrast to arthropod CISs or NBs, however, these NPs do not differentiate and immigrate in any recognizable spatio-temporally stereotyped pattern. Whereas one study claimed them to represent NSCs similar to hexapod NBs [76], this view was soon contradicted [20,159] since the onychophoran NPs divide morphologically symmetrically and neither their arrangement nor molecular data support similarities in the generation of NBs and onychophoran NPs. However, analysis of the distribution of neural cell fate determinants, such as *prospero*, that are known to be asymmetrically distributed in hexapod and branchiopod NB divisions [36,160] would further contribute to the characterization of the onychophoran NPs. Owing to the limited similarities to any arthropod taxon, the inclusion of the onychophoran out-group in evolutionary considerations on arthropod neurogenesis has so far proven less enlightening than hoped for. The plesiomorphic or apomorphic nature of early neurogenic processes in onychophorans (see [76] versus [20,159]) with respect to the panarthropod stem species remains still insufficiently resolved (Figure 17).

Interestingly, advanced onychophoran development also features structures that have been called ‘ventral organs’ and represent transformations of the initial neuroectodermal thickenings (Figure 15F) [158,161-163]. As in pycnogonids and myriapods, these ‘ventral organs’ represent segmentally arranged, multi-layered cell accumulations, which in onychophorans lie apical to the growing medullary cords. However, in contrast to the two arthropod taxa, the ‘ventral organs’ in the onychophoran trunk do not invaginate. After having reached notable dimensions at the apical surface, they suddenly atrophy during late development [65,158]. In adults, their only remaining signs are spatially restricted ‘ventral pits’ [164]. The exact function and fate of the ‘ventral organ’ cells has remained elusive in histological studies [161-163]. Recent *in vivo* cell proliferation experiments have revealed almost ubiquitous DNA synthesis in the columnar ‘ventral organ’ cells [165], and apoptosis has been shown during the subsequent decrease of ‘ventral organ’ extensions [158]. It is important to note, however, that observation of apoptosis in late ‘ventral organ’ stages does not automatically account for the fate of all cells that have been formed previously and that even with all new data, a function of the ‘ventral organs’ has still not emerged. Nonetheless, other authors [65,158] have recently rejected a previous claim by Pflugfelder [163] of an involvement of the ‘ventral organs’ in advanced neurogenesis. This rejection is mainly based on i) the already well-established axonal scaffold of the VNC at the time of ‘ventral organ’ differentiation and on ii) the spatial segregation of the apical ‘ventral organs’ from the more interior medullary cords. Obviously, the first argument in itself does not exclude continuing neurogenesis in more apical layers, as also exemplified in the present study. Similarly, the second argument does not necessarily speak against a neurogenic function. Apart from Pflugfelder [163], at least two additional studies [161,162] discuss and unmistakably depict persisting cell strands between the apically decreasing ‘ventral organs’ and the medullary cords. In one of these studies [161], it is even explicitly considered that cells may migrate along these strands into the medullary cords, thus contributing to the ‘ventral organ’ decrease (p. 21 in [161]). Since the new data [158] do not allow refutation of the previously documented cell strands, it remains feasible that they were missed. Therefore, we consider the rejection of a role of the onychophoran ‘ventral organs’ as sources for neural cell material during advanced development as being premature and in need of additional targeted investigations.

### The evolution of arthropod neurogenesis – two possible scenarios

Two possible scenarios of the evolution of arthropod neurogenesis are shown in Figure 17. They are set against the Chelicerata (Pycnogonida + Euchelicerata) and Mandibulata (Myriapoda + Tetraconata) hypotheses that again find support in several recent studies [23,51,55,79]. Based on a mapping of available data and comparison across the different taxa, supposed characteristic features of neurogenesis have been traced back to several key nodes of the tree. A colour-coding has been implemented in order to highlight whether specific features are apomorphic (green) or plesiomorphic (red), cannot be resolved (black) or are in need of reinvestigation and further clarification (grey with question mark). Instead of advocating a single ‘true’ scenario only, we purposefully depict two scenarios with different implications for the stem species pattern of arthropod neurogenesis (Figure 17A,B; grey boxes, respectively). As unsatisfactory as this may seem, it is still necessary in order to highlight the considerable persisting gaps in our available data on neurogenesis in non-model taxa. Despite the progress in the field during the last two decades, any reconstruction of the transformations of neurogenic processes during arthropod evolution remains to a certain extent debatable.

The first scenario (Figure 17A) presents a simple and conflict-free explanation of the specific features of pycnogonid neurogenesis by advocating convergent evolution of NSCs within pycnogonids and tetraconates. Contrary to any notions of ‘primitiveness’ or ‘ancestrality’ of extant sea spiders (in the sense of having retained plesiomorphic character states) that are frequently nourished, the neurogenic processes in crown-group Pycnogonida are here suggested to have been derived within the pycnogonid stem-lineage. This would render them useless for the reconstruction of the ancestral arthropod pattern.

The second scenario (Figure 17B) takes our considerable gaps of knowledge especially for advanced arthropod neurogenesis into account and results in the more intriguing proposal of NSCs already in the arthropod stem species. The plausibility of this view obviously hinges on the differentiation of NSCs during myriapod neurogenesis, which – as discussed above – remains to date neither conclusively proven nor clearly refutable.

## Conclusions

Embryonic neurogenesis of *Pseudopallene* sp. combines features of central nervous system development that have been hitherto described only separately in different arthropod taxa. This two-phase character of pycnogonid neurogenesis calls for a detailed reinvestigation of other non-model arthropods at the cellular level over the entire course of neurogenesis.

In order to meaningfully reconstruct and better understand the evolution of arthropod neurogenesis, it is of paramount importance to obtain additional data that are densely sampled for a broader range of taxa. These data need to include the involved cell types, their genealogical interrelationships (lineage studies) and the differentiating adult structures (neuron and glial types, neuropil architecture, etc.) in combination with molecular cell type characteristics and, ideally, functional analyses. For instance, in spite of the discussed differences between pycnogonid NSCs and tetraconate NBs, a potential common origin of both neural precursors from an ancestral NSC type cannot be excluded. To shed more light on this issue, not only further insights into the pycnogonid NSCs have to be gained, but also additional studies on advanced myriapod neurogenesis at the cellular level need to be conducted. Furthermore, the likely paraphyly of crustaceans makes more studies on non-malacostracan and non-branchiopod representatives pivotal in order to resolve tetraconate NB evolution in more detail and thus elucidate the plesiomorphic features of the tetraconate stem species.

## Abbreviations

ch: Cheliforal segment; con: Connective; EC: (neuro)Ectodermal cell; CIS: Cell internalization site; EPC: Epidermis cell; ES: Embryonic stage; GC: Ganglion cell; GMC: Ganglion mother cell; INP: Intermediate neural precursor; NB: Neuroblast; NSC: Neural stem cell; NP: Neural precursor; ov: Ovigeral segment; pa: Palpal segment; PH3: Phosphorylated histone H3; pp: Pedipalpal segment; pr: Proboscis; PS: Post-embryonic stage; VMR: Ventral midline region; VNE: Ventral neuroectoderm; x wl: Walking leg segment x; x wlg: Ganglion anlage of walking leg segment x.

## Competing interests

The authors declare that they have no competing interests.

## Authors’ contributions

GB and GS conceived the study and were involved in collecting and sorting the material. All authors helped to design the experiments. GB performed all laboratory work, analysed the data and wrote the first draft of the manuscript. AS and GS contributed laboratory space, reagents and analysis tools. All authors participated in the discussion of the results and contributed to the preparation of the final manuscript. All authors read and approved the final manuscript.
